# Unified Theory of Acceptance and Use of Technology Questionnaire in Swedish Prehospital Care: Translation, Cross-Cultural Adaptation, and Pilot Psychometric Study

**DOI:** 10.2196/96083

**Published:** 2026-09-08

**Authors:** David Summermatter, Jeanette Melin, Hans Blomberg, Björn Äng, Anneli Strömsöe

**Affiliations:** 1 School of Health and Welfare, Dalarna University, Högskolegatan 2, Falun, Sweden, 46 23 778000; 2 Department of Prehospital Care, Region Dalarna, Falun, Sweden; 3 Department of Leadership, Demand and Control, Swedish Defense University, Karlstad, Sweden; 4 Faculty of Health and Life Science, Linnaeus University, Kalmar, Sweden; 5 Department of Surgical Sciences/Anesthesiology and Intensive Care Medicine, University of Uppsala, Uppsala, Sweden; 6 Center for Clinical Research Dalarna, Region Dalarna, Uppsala University, Falun, Sweden; 7 Anesthesiology and Intensive Care, Department of Surgical Sciences, Uppsala University, Uppsala, Sweden; 8 Department of Anesthesia, Operating Departments and Intensive Care, Uppsala University Hospital, Uppsala, Sweden

**Keywords:** emergency medical services, questionnaires, surveys, attitude of health personnel, translation, cultural adaptation, cultural characteristics, psychometrics

## Abstract

**Background:**

Digital technologies in emergency medical services (EMS) have become crucial for patient outcomes and survival, and the integration of novel technologies drives rapid transformation within EMS. Understanding the acceptance and use of digital technology among EMS health care providers is paramount for implementation and effective use of novel digital tools. Currently, instruments assessing acceptance of technology are lacking within the Swedish EMS context.

**Objective:**

The aim of this study was to translate and culturally adapt the unified theory of acceptance and use of technology (UTAUT) questionnaire into Swedish for use in prehospital health care and to evaluate its psychometric properties.

**Methods:**

A methodological study was conducted using sequential qualitative and quantitative components, following the International Society for Pharmacoeconomics and Outcomes Research (ISPOR) guidelines for translation and cultural adaptation, and the Rasch Reporting Guideline for Rehabilitation Research (RULER) framework to guide the reporting of Rasch analysis. The translation process included forward and backward translation and harmonization, followed by a first round of cognitive interviews with ambulance nurses (n=6). A revised version was tested in the second round with the same participants. The adapted questionnaire was pilot tested among EMS personnel (n=91). Psychometric properties, including unidimensionality, response category functioning, targeting, reliability, and differential item functioning (DIF), were evaluated using Rasch analysis.

**Results:**

The cognitive interviews identified linguistic and contextual ambiguities requiring item revisions for several UTAUT constructs related to performance expectancy, social influence, and behavioral intention (BI). Rasch analysis revealed disordered response categories for several items, requiring collapsing of categories before analysis. After adjustment, overall item fit was acceptable, with minor misfit in a small number of items (facilitating conditions 1 infit mean square fit statistics [MSQ]=0.68; BI2 infit MSQ=0.57). Unidimensionality was supported for each UTAUT construct (Eigenvalues 1.59‐1.80), although reliability (person separation index range 0.55‐0.75) and targeting (person mean location 0.29‐0.74 logits) differed, with limited item coverage and measurement precision for the construct BI (1.24 logits). No substantial DIF for sex was identified.

**Conclusions:**

The translated and adapted Swedish UTAUT questionnaire achieved linguistic and contextual relevance among Swedish EMS personnel, although acceptable psychometric properties were not observed across all constructs, particularly the construct of BI. This indicates that translation and adaptation alone are not sufficient, and further refinement of the questionnaire with larger samples is warranted before routine and broader application can be recommended in Swedish prehospital care.

## Introduction

Digital technologies have been an integral part of emergency medical services (EMS), and novel digital technologies have been crucial for patient outcomes and survival [1-3]. Although telemedicine has been used for decades in EMS [4], the ongoing digitalization is transforming EMS [5]. Video consultation exemplifies digital technology used in ambulance health care, but its implementation in prehospital care remains challenging due to technological limitations, training needs, regulatory issues, and resistance among health care professionals [6].

In Swedish prehospital care, digital technologies, such as video consultation, are an emerging tool to enhance real-time communication between ambulance nurses and physicians, and influence patient safety and interprofessional collaboration [7,8]. This might increase patient involvement and contribute to better understanding and consensus regarding patient care. However, successful integration of digital technologies is influenced by health care professionals’ acceptance and willingness to use them [9-11]. Understanding the acceptance and use of novel technology among health care providers is paramount for implementation and the effective use of these novel digital tools [12].

Acceptance and willingness to use digital technology may be more challenging in a prehospital setting, as there are often stressful situations with time-critical conditions and a physically demanding work environment [13]. Independent patient assessment, advanced care, clinical interventions, and limited access to support on scene or during transport may affect how EMS personnel accept technology. In Sweden, ambulance nurses lead prehospital emergency care and have the primary responsibility for patient care. The Swedish EMS system is tax-funded and provides a nontiered, multipurpose Advanced Life Support (ALS) ambulance fleet, staffed with registered nurses and emergency medical technicians. Physicians are not routinely present in ambulance units, staffing mainly rapid response cars and air ambulances [9].

Theoretical models can provide support for understanding what makes health care professionals choose to accept digital technology. The prevailing models explaining acceptance of health care technology are the technology acceptance model (TAM) and the unified theory of acceptance and use of technology (UTAUT) [14-16]. While TAM mainly focuses on individual perception of ease of use and usefulness, it does not fully account for organizational factors [17]. Developed by Venkatesh et al in 2003 [18], the UTAUT merges 8 different earlier models to explain acceptance of technology, including TAM. UTAUT was developed to understand individual user acceptance in organizations associated with acceptance of new technology, making it suitable for the EMS environment, where technology is mandated by management. The UTAUT model identifies 4 key determinants related to behavioral intention (BI) and use behavior, that is, performance expectancy (PE), effort expectancy (EE), social influence (SI), and facilitating conditions (FC) [18].

The importance of culturally adapting questionnaires in health care research is well-established, and adaptation is recommended even for widely used questionnaires [19,20]. However, translation and adaptation go beyond linguistic equivalence. Although questionnaire items might be linguistically equivalent in countries sharing the same language, their meaning and interpretation might differ due to professional practices and terminology, workplace-related cultural norms, and health care system characteristics [20]. Without appropriate adaptation, instruments with poorer validity and measurement properties might emerge [21]. Ensuring that a translated questionnaire retains its construct validity and measurement properties requires evaluation beyond linguistic assessment. Rasch analysis provides a framework to investigate internal construct validity, rating scale functioning, and cross-cultural invariance [22].

Previous research has shown that both cultural and contextual adaptation are important when digital technology is to be used in time-critical environments within health care [23]. In previous studies, UTAUT has proven to be a useful model for understanding healthcare professionals’ use and acceptance of digital technology [14,24-26]. Prehospital health care is technology-intensive, and EMS personnel need to continuously stay up-to-date to be able to use available digital technology. Today, there is a knowledge gap in how EMS personnel’s acceptance and intended use of digital technologies. To begin to address this gap, there is a need to study EMS personnel’s use and acceptance of digital technology to inform its implementation in clinical practice. This study aimed to translate, culturally adapt, and perform an initial psychometric evaluation of the UTAUT questionnaire to evaluate acceptance and use of digital technology in a Swedish prehospital context.

## Methods

### Design

This study used a methodological design with sequential qualitative and quantitative components to translate, adapt, and evaluate the psychometric properties of the UTAUT questionnaire for use in a Swedish prehospital context. The research process consisted of three main phases as follows: (1) translation and cultural adaptation to the EMS context, including cognitive interviews; (2) pilot study; and (3) psychometric evaluation using Rasch analysis. Cognitive interviews included a combination of the think-aloud technique and verbal probing questions [27,28]. This approach was chosen to assess qualitative and quantitative aspects of content validity. Translation and cultural adaptation followed the International Society for Pharmacoeconomics and Outcomes Research (ISPOR) guidelines [29], to ensure conceptual equivalence and cultural relevance of the Swedish version of the questionnaire. Initial psychometric evaluation was reported in accordance with the RULER (Rasch Reporting Guideline for Rehabilitation Research) statement [30] (Checklist 1). This paper was prepared in accordance with the STROBE (Strengthening the Reporting of Observational Studies in Epidemiology) checklist [31] (Checklist 2).

### UTAUT

UTAUT was originally designed to explain and predict user acceptance of information technology in organizational contexts. The model combines 8 previously developed TAMs, including diffusion of innovation [32], the theory of planned behavior [33], the theory of reasoned action [34], and the TAM [17]. The UTAUT is widely used, considered to be a comprehensive model investigating acceptance of technology, and has demonstrated the ability to predict 70% of the variance in both use of technology and BI to use [18,26,35].

The UTAUT questionnaire contains 19 items and identifies four determinants that influence BI to use technology: (1) PE – defined as “the degree to which an individual believes that using the system will help him or her to attain gains in job performance” (4 items), (2) EE – defined as “the degree of ease associated with use of the system” (4 items), (3) SI – defined as “the degree to which an individual perceives that important others believe he or she should use the new system” (4 items), (4) FC – defined as “the degree to which an individual believes that an organizational and technical infrastructure exists to support use of the system” (4 items), and (5) BI, which refers to an individual’s intention to use the technology or system (3 items), is measured as a separate construct [18]. Of these constructs, 4 are moderated by demographic factors, such as gender, age, experience, and voluntariness of use.

In the original study, items were measured using a 7-point Likert scale. In this study, a 5-point Likert scale was used. This methodological deviation was deliberately made to enhance response quality and usability and to adapt to the prehospital context. Ambulance care is characterized by a demanding work environment, unpredictability, and being practiced in a dynamic and uncontrolled environment with high cognitive load and time-critical decision-making. These circumstances may reduce the willingness to respond to a larger number of response options, and the ability to identify fine distinctions between response categories might be reduced, meaning this would add no psychometric advantages [36]. This choice is further supported by previous research indicating that a 5-category scale provides a balance between measurement precision and response quality, compared with a larger number of response categories [36,37]. Items were scored on a 5-point Likert scale ranging from 1=“strongly disagree,” 2=“disagree,” 3=“neither agree nor disagree,” 4=“agree,” to 5=“strongly agree,” with higher scores indicating better endorsement of acceptance and use of technology. The inclusion of a neutral midpoint was considered advantageous to maintain comparability with the original UTAUT instrument and to offer respondents the opportunity to express context-dependent positions (eg, it depends). Although midpoint categories remain debated in survey methodology, in particular as regards concerns about central tendency bias, their inclusion was considered appropriate due to the hypothetical nature of technology in this context [38].

### Translation and Cultural Adaptation

The UTAUT questionnaire was translated and culturally adapted in accordance with the ISPOR guidelines [29], aiming to ensure cultural appropriateness and conceptual relevance for Swedish prehospital care. The steps according to the ISPOR guidelines included forward and backward translation, harmonization, and 2 iterative rounds of cognitive interviews. The process is summarized in Table 1.

**Table 1.** Translation and cultural adaptation process according to ISPOR^a^ guidelines^b^.

ISPOR step	Description of the step	Procedure in the study
Preparation	Permission and planning	Permission to use and translate the UTAUT^c^ was obtained from the original author and publisher (Society for Management Information Systems and Management Information Systems Research Center of the University of Minnesota).
Forward translation	Independent translation into the target language	Two researchers, both fluent in the source language and target language, translated the questionnaire from English into Swedish. One had expertise in anesthetics and EMS^d^ care; the other in EMS care.
Reconciliation	Merging translations into a single Swedish version	The two forward translations were merged into a single Swedish version. Disagreement between translators was resolved by consensus.
Backward translation	Translation back into the source language	An independent professional translator translated the Swedish version back into English. The translator was blinded to the questionnaire and unfamiliar with EMS and UTAUT.
Back translation review	Translation back into source language	The back-translated version was reviewed against the original UTAUT questionnaire to identify differences.
Harmonization	Resolution of discrepancies	Discussion about discrepancies among the researchers and the professional translator. Item rewording to ensure contextual relevance and linguistic accuracy.
Cognitive interviews round 1	Assessment of clarity, comprehensibility, and relevance	Active-duty ambulance nurses (n=6) participated in think-aloud interviews using probing questions to evaluate item clarity, interpretation, and response options.
Review of cognitive interviews round 1	Revision of questionnaire	Findings from round 1 were reviewed by the research team to revise item wordings and contextual alignment.
Cognitive interviews round 2	Evaluation of the revised version	A second round of think-aloud interviews with the same participants as in round 1 to reevaluate the revised questionnaire and assess the identified issues.
Proofreading	Linguistic review before pilot testing	The questionnaire was reviewed for grammar, terminology, and readability.
Final report	Documentation	Generation of a final, detailed, and comprehensive report, documenting all ISPOR steps.
Pilot testing^e^	—^f^	The adapted UTAUT questionnaire was pilot tested among ambulance nurses (n=91) to evaluate its psychometric properties.

a ISPOR: International Society for Pharmacoeconomics and Outcome Research.

b The translation and cultural adaptation process follows International Society of Pharmacoeconomics and Outcome Research (ISPOR) guidelines.

c UTAUT: unified theory of acceptance and use of technology.

d EMS: emergency medical services.

e Pilot testing is not part of the ISPOR guidelines, and this is a separate step.

f Not applicable.

### Participants

The study was conducted in 2 ambulance organizations, Region Kronoberg and Region Sörmland, located in the southern and east-central parts of Sweden. Region Kronoberg covers an area of 8457.96 km^2^ with a population of 203,351 inhabitants (2024) and provides ambulance services from 8 ambulance stations. Region Sörmland covers an area of 6097.20 km^2^ with a population of 301,542 (2024) [39] and provides ambulance services from 8 ambulance stations. The regions consist of a similar mix of suburban and rural areas, with population density varying substantially between larger towns and sparsely populated areas. Both EMS organizations offer a tax-funded EMS system providing a 24-hour nontiered, multipurpose, and all–ALS ambulance fleet staffed with 2-person crew configurations, either an emergency medical technician and a registered nurse or 2 registered nurses. In addition, the EMS organization in Sörmland operates nurse-staffed single responder vehicles for assessment and triage, although this additional resource does not replace ambulance units. Furthermore, this organization has access to video consultation at the dispatcher level for selected patients. This function is not available for ambulance crews. The organizations were selected as they represent typical Swedish EMS organizations where digital solutions, such as video consultation, are under discussion for implementation in the future.

#### Participants for Cognitive Interviews

The inclusion criteria were active-duty ambulance nurses within the EMS organization with a variation in sex and age to capture a broad range of perspectives. The recommendations based on ISPOR guidelines determined the number of participants that would constitute the sample (5‐8 respondents) [29].

#### Participants for Pilot Psychometric Evaluation

The inclusion criteria were EMS personnel employed by the participating organizations. A census sampling approach was used, where the final sample reflects those who chose to respond. Demographic information collected included gender, age, years of experience in ambulance care, professional occupation, and previous experience with video consultations in health care. Participants in the cognitive interviews were ineligible for the main survey and were therefore excluded from data collection. The sample size was evaluated against published recommendations for Rasch analysis. Previous methodological studies suggest that samples of 50 to 100 participants can provide stable and informative item calibrations in exploratory and pilot Rasch studies, depending on targeting and desired precision. Therefore, the achieved sample of 91 participants was considered adequate for the psychometric evaluation and Rasch analyses performed in this pilot study [40-42].

### Data Collection

#### Cognitive Interviews

Data collection was conducted in 2 rounds. The first round took place between November 7 and November 20, 2024, and the second round between December 30, 2024, and January 14, 2025. Potential participants were identified by the EMS directors of the 2 ambulance organizations at the request of the research team, who subsequently contacted the participants by email and invited them to participate in the study. After the participants responded that they wanted to be part of the study, their names were provided to the research team. Each potential participant was contacted individually by email and invited to participate in a cognitive interview. Consent was given when participants agreed to participate in the interview. Written information about the study was provided during questionnaire distribution. Interviews were conducted online using Microsoft Teams, recorded as video and audio, and transcribed verbatim. All recordings were downloaded and securely stored on a university research server and deleted from the Teams cloud to ensure data protection. The research team had no control over the selection of these individuals. A semistructured interview guide based on a combination of “think-aloud” and verbal probing questions was used [27,28]. Cognitive interviews were conducted in 2 iterative rounds to assess the conceptual equivalence and comprehensibility of the culturally adapted UTAUT questionnaire. These can be found in the Multimedia Appendix 1. Round 1 aimed at identifying ambiguities, interpretation, and translation issues. The second round aimed to evaluate the effectiveness of the revision by resolving issues identified in round 1. The same participants were interviewed in both cognitive interview rounds, allowing them to provide feedback on revisions and whether previously identified issues had been addressed. The participants were asked to complete the questionnaire online and to comment on each item with a focus on understanding, clarity, their interpretation of terms, and relevance. Each item was presented separately, with no possibility for the participants to see the entire questionnaire. This allowed participants to describe their understanding of each item and to identify wording that was unclear, ambiguous, or inappropriate for the prehospital context. Probe questions focused on comprehension, judgment, and suitability of response options.

#### Pilot Psychometric Evaluation: Evaluation Process of the Questionnaire

The data collection was conducted from February 13, 2025, to May 1, 2025. The participants in the pilot study were recruited from the 2 different ambulance organizations. A total of 357 employees were invited to participate in the study. The participation was voluntary. Questionnaire data were collected using an online survey tool hosted on a secure platform by Dalarna University. To facilitate questionnaire distribution, a survey link was distributed by the EMS directors to the entire ambulance organization. During data collection, the research team sent 2 reminder emails, and one of the research team members visited every ambulance station of both ambulance organizations to remind participants to complete the questionnaire and to answer questions raised during the data collection period. During the visits, a flyer with a QR code was distributed to remind participants to complete the questionnaire. Participants in the pilot study had the opportunity to provide free-text comments on each questionnaire item.

### Data Analysis

#### Cognitive Interview Analysis

Interview transcripts were analyzed using charting to enable structural item-by-item synthesis. Each participant’s response was entered in a predefined Microsoft Excel spreadsheet organized by questionnaire item. The responses were then summarized item-by-item and across participants to identify issues or ambiguities. Ambiguities were not treated uniformly; issues reported by multiple participants or impacting the conceptual equivalence were prioritized for revision. Minor linguistic issues were addressed later in the refinement process. Items identified as problematic and suggested for revision by participants were reviewed by the research team, and discussions were held to reach consensus about adjustments and modifications to improve clarity and conceptual equivalence. The revised version was then tested in a second round of cognitive interviews with the same participants from the first round of cognitive interviews. The purpose of the second round was to assess if previously identified issues had been resolved and to confirm contextual relevance and clarity of the revised items.

#### Pilot Psychometric Evaluation Analysis

The psychometric evaluation of the translated and culturally adapted questionnaire was conducted using Rasch analysis based on the partial credit model to evaluate the measurement properties of each UTAUT subscale. Rasch analysis was performed using the *easyRasch* package [43] in the R statistical computing environment (version 4.5.2; R Core Team), including its dependency packages, which for the Rasch analysis are *eRm* [44], *iarm* [45], *mirt* [46], and *psychotree* [47]. Differential item functioning (DIF) was assessed using *easyRasch* to test for statistical differences and RUMM2030 software (RUMM Laboratory Pty LTD) [48] to obtain numerical DIF estimates and graphical displays to support interpretation and verification of DIF, particularly if statistical tests in *easyRasch* indicated no significant DIF. The *easyRasch* package required a minimum of 3 responses to generate stable threshold estimates [43]. All questionnaire items were mandatory, and all participants (n=91) provided complete response data and were included in the analysis with no missing data.

Unidimensionality was assessed using fit-statistics, assessment of local independence, and principal component analysis of residuals. The interpretation item fit was primarily based on simulation-based thresholds provided by the *easyRasch* package and derived from the actual sample size and scale characteristics [49]. Local dependency was assessed by evaluating the simulation-based cutoffs derived under the Rasch model provided by *easyRasch* [43].

When assessing principal component analysis of residuals, Eigenvalues below 1.5 are commonly considered supportive of unidimensionality [50]. However, given the relatively small sample size of 91 and limited items (3-4) per subscale, this rule of thumb might be overly restrictive. In this study, an Eigenvalue of below 2.0 was considered an acceptable indication of unidimensionality. Ordered response category properties were assessed using item characteristic curve plots and threshold maps. DIF in this study was used only for exploratory purposes, due to the limited sample size (n=91), which could reduce the statistical power to detect meaningful, reliable group-related differences [51]. Mean item location values within ±0.5 were considered well targeted [22,40]. Reliability was primarily assessed by reporting the person separation index (PSI) and Cronbach α, and secondarily by weighting the likelihood estimate by relative measurement uncertainty (WLE-RMU) [52].

### Ethical Considerations

This study was approved by the Swedish Ethical Review Board (Dnr 2024-03401-01). All procedures and data processing were in line with the General Data Protection Regulation and the Declaration of Helsinki [53]. Participation was voluntary, and all participants for the cognitive interview received written and verbal information with the possibility to withdraw from the study without consequence. Informed consent was obtained by the participants electronically clicking “Agree to participate” to proceed to the questionnaire or “Disagree” to refuse to participate. Data collected from the UTAUT survey were submitted anonymously by the participants. To maintain anonymity, no identifying data were collected; each participant was allotted a unique nontraceable code. The participants did not receive any financial or other form of compensation for their participation in the interviews or the pilot study.

## Results

### Translation and Cross-Cultural Adaptation

#### Cognitive Interviews

A total of 6 active-duty ambulance nurses, 3 from each EMS organization, participated in the cognitive interviews. Most were men (n=5), and the ages ranged from 34 to 63 (mean 47, SD 10.6) years. The interviews in round 1 lasted between 36 minutes and 81 minutes, and those in round 2 lasted between 30 and 51 minutes.

#### Round 1 Finding

##### Overview

In the first round, individual interviews were conducted in which participants were asked to provide their views on the assessment of the conceptual equivalence and comprehensibility of the culturally adapted UTAUT questionnaire. The model was clarified and adapted to the theoretical model and the 4 constructs in UTAUT (Table 2).

**Table 2.** Issues identified during round 1 of cognitive interviews and findings from round 2 of cognitive interviews.

Construct	Item^a^ in Swedish	Findings in round 1	Item^a^ after revision	Findings in round 2
Performance expectancy 3	Using digital technology (such as video consultations) increases my productivity.	The term “productivity” was perceived as confusing.	Video consultation will help increase availability for new ambulance assignments.	Improved clarity with no further ambiguities identified.
Effort expectancy 1	My commitment towards digital technology (such as video consultations) should be clear and understandable.	The statement was unclear.	It will be clear and easy to understand how I should use video consultation.	Item is well understood.
Social influence 1	I am influenced by others to use the system.	The term “system” was used, and the item was perceived as unclear.	People I work with will influence me to use video consultation.	Refined terminology with no ambiguities.
Social influence 2	People who are important to me think that I should use digital technology (such as video consultation).	“Important people” were considered to refer to the prehospital working context.	People I work with will think I should use video consultation.	Revision aligned with the intended context.
Social influence 3	The management is helpful in the use of digital technology (such as video consultations).	The term “helpful” was perceived as confusing.	The management will be helpful when I start using video consultation.	No interpretation issues.
Facilitating conditions 1	I have the resources needed to use digital technology (such as video consultations).	Difficulties in understanding “resources,” which were interpreted differently by different people.	I will have the necessary resources to use video consultations.	Better understanding.
Facilitating conditions 3	Digital technology (eg, video consultation) is not compatible with other systems I use.	Inconsistent terminology: “digital technology” should be replaced by “video consultation.” Item had negative wording, which led to ambiguities.	Video consultation will be compatible with other digital systems I use.	No interpretation difficulties were observed after the removal of negative wording.
Facilitating conditions 4	A specific person is available to assist with system issues.	As mentioned above, the word “system” should be replaced in this item.	Technical support will always be available when needed.	Item interpreted as intended.
Behavioral Intention 1	I intend to use digital technology (eg, video consultation) in the longer term.	The meaning of “in the longer term” is too ambiguous.	Provided that the organization implements video consultation, I intend to use it within a year.	Clear understanding of the time frame.
Behavioral intention 2	In the longer term, I plan to use digital technology (eg, video consultation).	The term “in the longer term” was too ambiguous.	Provided that the organization implements video consultation, I will use it as soon as it becomes available.	Clear understanding of the time frame.

a Items have been translated from Swedish into English. A Swedish version is available in Multimedia Appendix 1.

##### PE

After the first round of cognitive interviews, participants generally demonstrated a clear understanding of items related to PE. Participants associated the items with the perception of usefulness and benefits of digital technology in ambulance care. The statements in items PE1 and PE4 were perceived as clear and understandable. However, the term “digital technology (such as video consultation),” which was used throughout the questionnaire, led to ambiguity about what was investigated—digital technology or video consultation? Furthermore, item PE3 caused confusion among the participants regarding the meaning of the term “productivity.” The statement was clear but open to interpretation, and participants suggested clarification to reduce uncertainty. These findings suggested a need to clarify the technological context and the definition of the term “productivity” in ambulance care.

##### EE

EE items were perceived as relating to the ease of learning to use digital technology in daily work. Think-aloud data indicated that 1 item (EE1) needed revision, whereas EE2, EE3, and EE 4 were perceived as clear and understandable. For item EE1, almost all participants repeated the statement several times to try to understand the meaning of “my commitment,” which was translated from “my interaction” in the original version. During the interviews, participants received access to the original UTAUT item (EE1) for better understanding (“My interaction with the system would be clear and understandable”). These findings indicated a lack of conceptual equivalence for item EE1 and necessitated a revision.

##### SI

By contrast, items for SI were understood as relating to peer influence, contextual norms, and organizational support rather than social norms. This constitutes a context-related conceptual deviation from the original meaning in the UTAUT. Item SI1 required a revision in its wording after feedback from participants. Instead of using “system” in the statement, participants suggested using the word “video consultation.” Item SI3 used the term “supportive” and was generally perceived as referring to help in general, a resource, or organizational help. However, 1 participant considered this word broad and imprecise. Overall, participants interpreted people who were influencing them (SI1) and people who were important to them (SI2) primarily as coworkers and senior management, rather than close family and relatives.

##### FC

Participants interpreted the items for FC as relating to a combination of training, individual competence, technical infrastructure, and organizational support. Generally, items FC1-FC4 revealed issues with the wording “digital technology” (such as video consultations). Participants suggested a clearer definition for items FC3 and FC4, and to use the wording “video consultation” for system problems, not only for items in FC, but in the whole UTAUT questionnaire. Furthermore, in the original UTAUT version, FC1 states, “I have the resources necessary to use the system.” In this version, the word “resource” was translated into Swedish and was perceived by participants as difficult, leading to different interpretations depending on the context, with a main focus on technical solutions and logistics. Item FC3 used negative wording in the statement. Participants perceived this as increasing cognitive load and suggested this item should be revised.

##### BI

BI items were generally interpreted as relating to the participant’s willingness to use digital technology in the future, and participants understood the items well. However, in the translated version, item BI1-BI3 uses the term “in the longer term” instead of a categorical number as in the original UTAUT. This contextual choice by the research team led to ambiguities in interpreting the items, causing uncertainty in distinguishing between actual opportunities and future intentions. Participants indicated that a clear time frame would facilitate interpretation of the items.

### Round 2 Findings

#### Overview

Round 2 cognitive interviews were conducted 6 weeks after the first round of cognitive interviews. Based on the results from round 1, revisions were made to items with interpretation problems. This revision aimed to clarify and align wording more closely to the theoretical model and constructs of UTAUT. Findings indicated an overall improved clarity and conceptual alignment across all constructs. Issues identified in round 1 were mostly resolved, and no new systematic comprehension problems emerged during round 2 (Table 2).

#### PE

Findings in round 2 indicated that the revisions made after round 1 clarified the technological context by using the term “video consultation” throughout the questionnaire. The term “productivity” in item PE3 was replaced by “availability of ambulance care”; participants could relate the term to available ambulance resources, logistics, and geographical location. No further ambiguities were identified, and the other items were considered satisfactory.

#### EE

Following the revision of item EE1, the item wording changed from “my commitment” to “clear and understandable.” Participants interpreted this as self-managed, smooth use of technology, and clear guidelines. This change created a minor conceptual nuance shift but retained the main concept of perceived ease of use. The item was therefore retained for further evaluation. Items EE2-EE4 were perceived as clear and understandable, and no additional issues were identified. To enhance readability and linguistic fluency, the research team made minor grammatical adjustments.

#### SI

Items investigating SI underwent a shift in interpretation after revision. In round 1, participants related the items to coworkers and workplace-related colleagues. Based on the participants’ feedback, items SI1 and SI2 were reformulated to enhance contextual collegial relationship (“People I work with”). This adaptation aimed to reflect the workplace orientation described by the participants during cognitive interviews in round 1, focusing only on professional relationships. Item SI4’s wording was revised and aligned more with the original UTAUT item by specifying the context of prehospital care. Probing questions revealed that the participants interpreted “organization” as the ambulance service and justified a contextual clarification of the item.

#### FC

In round 2, participants demonstrated a better understanding of the FC items after revision. For item FC1, the word “resource” was replaced by “conditions.” Responses to the probe questions revealed that participants interpreted the new word as both individual and organizational conditions for using video consultations. After the removal of the negative wording in FC3, this item was considered clear and understandable by the participants. Think-aloud and probing data showed that participants understood the item as referring to technical compatibility and integration of the system with existing health care systems.

#### BI

Findings in round 2 indicated improved clarity and better differentiation regarding future intentions to use video consultations. Based on the responses from round 1, the wording “*in the longer term*” in items BI1-BI3 was replaced by a specific time frame (“within a year”) and the assumption that video consultation would be implemented by the organization. This clarification shifted the focus among the participants to the intention-related wordings “*I intend*” (BI1), “*I predict*” (BI2), and “*I plan*” (BI3). “*I intend*” was interpreted as a general intention to use video consultations, whereas “*I predict*” was interpreted as an expectation to use video consultations, and “*I plan*” was understood as a more concrete plan to use the technology. Some participants reported some overlap between “*I intend*” and “*I predict*.”

### Pilot Psychometric Evaluation

#### Evaluation Process of the Questionnaire

A total of 357 employees were invited to participate in the study, of whom 94 completed the questionnaire. Of these, 91 met the eligibility criteria and were included in the analysis, resulting in an inclusion rate of 25.5%.

#### Rasch Analysis

A total of 91 ambulance nurses participated in this study and were included in the final analysis. The 6 ambulance nurses involved in the cognitive interviews did not complete the questionnaire. The demographic characteristics of the participants are presented in Table 3. The majority of participants were female (50/91, 55%), and a large share of participants had no previous experience with video consultation, although a few reported limited or extensive experience with video consultations.

**Table 3.** Participant characteristics of the questionnaire pilot study.

Characteristic	Participants (n=91)
Age (years), median (IQR)	41 (35-47)
Sex, n (%)	
Female	50 (55)
Male	41 (45)
Occupation, n (%)	
Registered nurse in active duty	86 (95)
Other^a^	5 (5.5)
Years of working experience in prehospital care (y), median (IQR)	9 (5-14)
Earlier experience with video consultations, n (%)	
No experience	72 (79.1)
Limited experience	17 (18.7)
Extensive experience	2 (2.2)

a Other occupation: registered nurses with other specializations or part-time management tasks.

**Figure 1.**
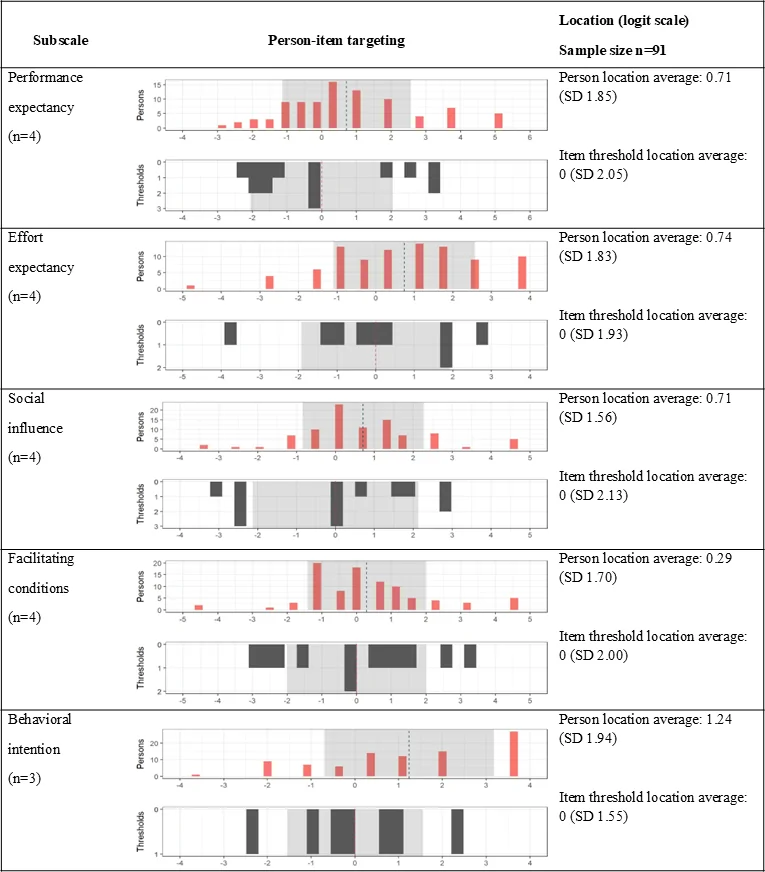
Person-item map for distributions of person abilities relative to item locations on the logit scale for each unified theory of acceptance and use of technology subscale.

The initial Rasch analysis of the instrument, with all items, revealed several disordered response categories and did not function as intended. Some response categories had very few or incomplete data for several items. To address this issue, those response categories were collapsed, and all the items were reanalyzed. Response categories for the items EE2-EE4, FC2, BI1, and BI2 were collapsed into 3 categories, while response categories for the items PE1-PE3, EE1, SI1-SI4, and FC1-FC3 were collapsed into 4 categories. The only remaining items with 5 response categories were PE2 and FC4. Results presented in this section refer to the dataset with reordered and collapsed items, where each construct of the UTAUT was analyzed separately. Table 4 presents an overview of Rasch analysis results for each UTAUT subscale after collapsing response categories, whereas Figure 1 provides a person-item map for distributions of person locations relative to item locations on the logit scale for each UTAUT subscale. Item-level fit statistics are presented in Table 5, and a summary of the cognitive interviews and Rasch analyses findings is presented in Table 6. Detailed information on response category curves, item thresholds, and targeting for each subscale is provided in the Multimedia Appendix 1.

**Table 4.** Overview of Rasch analyses results for each UTAUT^a^ subscale after collapsing response categories.

Construct	Items (n)	Infit MSQ^b,c^	Dimensionality (1st contrast eigenvalue)	Local dependency (residual correlations)	Response category functioning	Targeting^d^	DIF^e^	PSI^f,g^	Coefficient α^h^, (95% CI)	Notes
PE^i^	4	No misfit	1.59	Not detected	PE1 disordered	Acceptable	None	0.75	0.77 (0.70-0.84)	—^j^
Effort expectancy	4	No misfit	1.68	Not detected	Ordered	Acceptable	None	0.68	0.76 (0.67-0.84)	—
SI^k^	4	No misfit	1.79	No substantial local dependency	Ordered	Acceptable	None	0.64	0.71 (0.62-0.80)	One item pair residual correlation (SI1-SI2) exceeded the simulation-based cutoff (0.12).
FC^l^	4	FC1 overfit	1.80	Not detected	Ordered	Good	None	0.63	0.76 (0.68-0.83)	FC1 showed slight overfit (infit MSQ=0.68)
BI^m^	3	BI 2 overfit	1.77	Not detected	Ordered	Poor	None	0.55	0.80 (0.73-0.87)	BI2 showed slight overfit (infit MSQ =0.57). Person mean location 1.24, exceeding the mis-targeting threshold of 1.0 logit

a UTAUT: unified theory of acceptance and use of technology.

b MSQ: mean square fit statistics.

c Infit MSQ “No misfit” indicates that no individual items within the construct showed misfit based on Rasch item-fit statistics criteria for UTAUT subscales presented in Table 5.

d Targeting metrics (person location mean and SD) are presented separately in Figure 1.

e DIF: differential item functioning.

f PSI: person separation index.

g PSI values were calculated using Rasch analysis with the Partial Credit Model (n=91).

h Cronbach’s alpha is reported as a complementary measure of internal consistency.

i PE: performance expectancy.

j Not applicable.

k SI: social influence

l FC: facilitating conditions.

m BI: behavioral intention.

**Table 5.** Rasch item-fit statistics for UTAUT^a^ subscales^b^.

Subscale	Item^c^	Infit MSQ^d,e^	Infit difference	Relative location
PE^f^ (n=4)				
PE1	Video consultation will be useful in my work	0.729	No misfit	−1.48
PE2	Using video consultation will enable me to perform tasks more quickly within ambulance care	0.932	No misfit	−0.22
PE3	Video consultation will contribute to increased availability for new ambulance assignments	1.070	No misfit	−0.25
PE4	Using video consultation will improve the quality of care provided to patients	1.242	No misfit	−1.06
EE^g^ (n=4)				
EE1	It will be clear and understandable how to use video consultation	0.974	No misfit	−0.50
EE2	It will be easy for me to become a skilled user of video consultation	0.977	No misfit	0.21
EE3	I expect video consultation to be user-friendly	1.250	No misfit	−1.62
EE4	Video consultation will be easy for me to learn	0.667	No misfit	−1.18
SI^h^ (n=4)				
SI1	People I work with will influence me to use video consultation	0.969	No misfit	−0.55
SI2	People I work with will think that I should use video consultation	0.889	No misfit	−0.31
SI3	The management will be supportive when I begin using video consultation	1.166	No misfit	−1.00
SI4	Overall, the ambulance service will support the use of video consultation	0.966	No misfit	−0.99
FC^i^ (n=4)				
FC1	I will have the necessary conditions to use video consultation	0.683	0.019	−0.72
FC2	I will have the knowledge necessary to use video consultation	1.022	No misfit	−0.23
FC3	Video consultation will be compatible with other digital systems that I use	0.837	No misfit	−0.11
FC4	Technical support will always be available when needed	1.333	No misfit	−0.14
BI^j^ (n=3)				
BI1	Provided that the organization implements video consultation, I intend to use it within one year	1.277	No misfit	−0.87
BI2	Provided that the organization implements video consultation, I will use it as soon as it becomes available	0.574	0.083	−1.46
BI3	I plan to actively use video consultation in my daily work after it has been implemented in the organization	1.176	No misfit	−1.35

a UTAUT: unified theory of acceptance and use of technology.

b Analysis based on the partial credit model; n=91 complete cases.

c Items have been translated from Swedish into English. A Swedish version is available in Multimedia Appendix 1.

d MSQ: mean square fit statistics.

e Simulation-based thresholds were used for evaluating item fit.

f PE: performance expectancy.

g EE: effort expectancy.

h SI: social influence.

i FC: facilitating conditions.

j BI: behavioral intention.

**Table 6.** Summary of cognitive interview and Rasch analyses findings.^a^

Item (cognitive interviews)^b^	Cognitive interview results (round 2)	Results Rasch analysis (item level or construct level)^c^	Interpretation
PE^d^			
PE3: “Accessibility of ambulance care” instead of “productivity”	Relating the term to available ambulance resources, logistics, and geographical location.	Items PE1, PE3, and PE4 required the collapsing of response categories. PE1 still had disordered response category thresholds after collapsing.	Item revision enhanced comprehension but shifted the item toward an organizational interpretation of productivity. Category collapse reflects sparse responses and challenges in distinguishing between response categories.
EE^e^			
EE1: “Clear and easy to understand” instead of “my commitment”	Item interpreted as self-managed, smooth use of technology, and clear guidelines. Minor conceptual nuance shift but retained the concept of ease of use.	EE items showed ordered response category thresholds and no item misfit.	Item revision enhanced comprehension and preserved the core concept of EE. Stable response thresholds indicate participants’ ability to distinguish between response options.
SI^f^			
SI1-SI2: “People I work with” instead of “other people” and “important people around me.”	Participants interpreted items as professional or collegial influence. “*Organization*” was understood as an ambulance service.	SI items showed ordered response category thresholds and acceptable fit.	Item revision enhanced contextual specificity. “Important others” is a broad term and is interpreted by participants as important people in a workplace-centered sense.
FC^g^			
FC1: “Conditions” instead of the Swedish term “resource”	“*Conditions*” were interpreted as both individual and organizational conditions.	FC items had an overall acceptable fit, with a minor misfit in FC1.	Item revision enhanced clarity and indicated a broader understanding of individual and organizational support.
FC3: Positive instead of negative wording	After the removal of negation, the item was perceived as clear and was associated with technical integration with existing systems.	FC items had ordered response category thresholds.	The rewording of item FC3 led to reduced cognitive load. Rasch analysis indicated stable response category thresholds.
BI^h^			
BI1-BI3: Time frame and intention wording	Clarified time frame with intention verbs, although some semantic overlap remained.	BI subscale items indicated poor targeting and misfit in item BI2. Ordered response category thresholds.	Revision enhanced comprehension and shifted focus from time frame to intention verbs. Still, semantic overlaps remained, and misfit might be driven by poor targeting and ceiling tendencies.

a Findings based on two rounds of cognitive interviews (n=6) and subsequent Rasch analyses (n=91).

b Cognitive interview findings represent item-level interpretations and item-specific wording revisions identified during the adaptation process.

c Item-level Rasch results include response category functioning and item fit evaluation related to response category functioning and item fit.

d PE: performance expectancy.

e EE: effort expectancy.

f SI: social influence.

g FC: facilitating conditions.

h BI: behavioral intention.

#### PE

The PE subscale showed acceptable fit to the Rasch model (Table 4 and Multimedia Appendix 1). Unidimensionality was supported, with Eigenvalues within acceptable limits and residual correlations showed no substantial local dependency with a cutoff of 0.116. One item (PE1) showed a disordered threshold despite collapsing, which suggests that participants had a limited ability to distinguish between response categories 1 and 2. Item locations covered a reasonable range of the latent trait with a mean person location of 0.71 (SD 1.85), indicating acceptable targeting. No statistically significant DIF was found. The WLE-RMU was 0.811 (95% highest density credible interval [HDCI] 0.756-0.864) with 1000 iterations.

#### EE

The EE subscale demonstrated an acceptable fit to the Rasch model. Unidimensionality was supported, with no indication of local dependency (Table 4 and Multimedia Appendix 1). Thresholds were ordered, and targeting was acceptable, with a mean person location of 0.74 (SD 1.83). No DIF across sex (female vs male) was reported, and the WLE-RMU was 0.775 (95% HDCI 0.709-0.837).

#### SI

SI showed an acceptable fit to the Rasch model. Unidimensionality was supported, and residual correlation did not indicate substantial local dependency (Table 4 and Multimedia Appendix 1). Thresholds were ordered, suggesting the appropriate functioning of response categories. Targeting was acceptable with a mean person location of 0.71 (SD 1.56). No DIF across sex was detected, and the WLE-RMU was 0.734 (95% HDCI 0.659-0.806).

#### FC

The FC subscale demonstrated an overall acceptable fit to the Rasch model (Table 4 and Multimedia Appendix 1). One item (FC1) demonstrated overfit, but this was considered minor and did not affect the overall measurement properties of this subscale. The unidimensionality was supported, and no indication of local dependency was observed. Good targeting was reported with a mean person location of 0.29 (SD 1.70). The WLE-RMU was 0.775 (95% HDCI 0.708-0.836).

#### BI

The BI demonstrated an acceptable overall fit to the Rasch model (Table 4 and Multimedia Appendix 1) and only 1 item (BI 2) showed overfit. However, this was isolated, did not affect unidimensionality, and no local dependency was reported, indicating no practical impact on the overall measurement properties of this subscale. Targeting was poor, and the mean person location exceeded the threshold (mean 1.24, SD 1.94). The WLE-RMU was 0.752 (95% HDCI 0.678-0.822).

Overall, the findings from the cognitive interviews and Rasch analyses indicated that the item revisions generally improved clarity and understanding. The UTAUT constructs were associated with acceptable Rasch model requirements. However, disordered response category thresholds and item misfit were observed, particularly in the construct’s PE and BI constructs. These findings suggest that cognitive interviews identified interpretation and wording issues, while Rasch analyses provided additional measurement properties information of the adapted questionnaire.

## Discussion

### Principal Findings

This study aimed to translate, culturally adapt, and psychometrically evaluate the UTAUT questionnaire into Swedish in accordance with the ISPOR guidelines, complemented by Rasch analysis, to provide supporting evidence consistent with conceptual equivalence. Overall, the iterative translation process yielded improved item linguistic clarity and contextual relevance, but in some cases may have affected the original construct of the UTAUT. Psychometric evaluation revealed that some adapted items showed stable measurement properties, whereas others had disordered thresholds, required response category collapsing, and suggested poor targeting. Conceptual translation might increase contextual understanding and cognitive accessibility of questionnaire items, but could at the same time challenge construct integrity. While the findings from cognitive interviews suggested that participants understood the items as intended, the psychometric findings identified further refinement and evaluation. The small sample size has limited the ability to detect item misfit, and the results from the Rasch analyses should be interpreted with caution [49]. This psychometric evaluation provides initial insights and warrants further refinements of the questionnaire on acceptance of technology in Swedish EMS organizations.

### Comparison With Previous Work

#### PE

Cross-cultural adaptation for PE goes beyond mere linguistic translation, as cultural variation might influence interpretation. Item revision improved comprehension but shifted the focus of 1 item from individual productivity to organizational resource availability. The term “productivity” is seldom used in everyday EMS clinical practice and might suggest a performance indicator, not acknowledged as individual performance, but rather as system performance [54]. Although the 2 concepts are related, aspects like compliance with structured patient assessment and management, adherence to treatment protocols, and response times could be associated with productivity, especially if the individual actions of an ambulance nurse directly impact those factors [55]. However, these aspects reflect a broader performance dimension that includes teamwork, system efficiency, and resource availability [56]. This could suggest that the availability of an EMS system might capture individual performance and productivity in a broader sense. Nonetheless, an accurate definition of productivity in the health care context remains elusive [57]. The need for response category collapsing, due to sparse response distribution, may indicate that participants experienced difficulties in distinguishing between different response options or a broader conceptual interpretation of the construct PE. This suggests that professional terminology and the operational context of EMS influence how questionnaire items are perceived and interpreted [58-60].

#### EE

Item revision for the construct EE enhanced comprehension and preserved the core concept of ease of use. Nevertheless, a subtle nuance shift occurred in 1 item (EE1), which might alter the interpretation of the ease of technology use. The interpretation shifted from perceived individual user effort to individual and system usability, with clear guidelines on clinical use. In the context of EMS, this suggests that clear and understandable interaction with video consultation is associated with effortless use within clear clinical application guidelines, which is supported by Candefjord et al [11], emphasizing clear clinical protocols and system design for effortless use in clinical settings. However, both the original and the revised version emphasized the anticipatory expectation of ease and usability [18].

#### SI

For SI, the contextual adaptations increased the specificity of “*important others*” to professional peers and members of the organization. In the Swedish version, the wording “*persons with whom I collaborate*” was used in both items SI1 and SI2 to capture important members of the social group at work, which is in line with the concept of the UTAUT. Although not explicitly defined in the original UTAUT questionnaire, underlying theories of the UTAUT define important people and individuals who influence others as workplace-related reference groups [18,26,61]. Narrowing this group to professional peers might have contributed to acceptable measurement properties supported by Rasch analysis, suggesting the adequacy of contextual adaptation. However, the small residual correlation between items SI1 and SI2 may be related to the shared wording in these items, rather than to a violation of the unidimensionality of the construct.

#### FC

The FC items were affected by linguistic adjustments, which expanded the interpretation to individual and organizational conditions, and away from the initial interpretation related to logistics. Item FC1 showed an overfit in infit statistics, which may indicate a more predictable response pattern than expected by the model. This might reflect the linguistic adaptation after 2 rounds of cognitive interviews. The removal of negative wording in item FC3 reduced the cognitive load and might have contributed to response category functioning. Negatively worded items can introduce response bias, additional variance, and violate unidimensionality because negative statements tend to be easier to reject than positive ones [62]. However, the revision to a positively worded item was based on feedback in the cognitive interviews and was not guided by Rasch analysis.

#### BI

The BI item revisions focused on timeliness. In the original UTAUT instrument, BI items include a specific time frame (“I plan to use the system in the <n> month”). As video consultations have not yet been widely implemented in Swedish EMS, the phrasing “in the future” was used to maintain conceptual equivalence. However, this replacement was perceived by respondents as too ambiguous and as redundant temporal wording. Even carefully translated UTAUT statements may not accurately reflect participants’ perception of literally translated items [63]. Instead, and in line with published UTAUT adaptations [64], where a target technology is not yet implemented, alternative formulations were developed to maintain conceptual equivalence while ensuring contextual relevance for EMS. These adaptations were framed for the intention to operationalize video consultations without requiring immediate availability [65-67]. Cognitive interviews guided by the ISPOR principles proved to be essential to ensure semantic and conceptual equivalence, enhancing the content validity of the adapted UTAUT questionnaire for this specific context [29]. The poor targeting may reflect a ceiling effect and that the intention to use video consultation may already be high in this sample of medical professionals, as it was reported earlier in a Swedish primary care context [68]. Item misfit may be related to semantic overlap between items and may lead to a redundant response pattern.

### Implications

#### Implication for Cross-Cultural Adaptation

An important methodological insight from this study is that conceptual translation might enhance linguistic clarity and contextual appropriateness but may not necessarily preserve psychometric construct properties. The kind of potential discrepancy between conceptual equivalence and construct validity seen in this study has been reported earlier in cross-cultural adaptation in prehospital care [69], highlighting the complexity of cross-cultural adaptation in health care, and that methodological choices made during the translation process can significantly impact construct validity [70].

The approach to combine cognitive interviews with Rasch analysis in a translation process demonstrates how qualitative and quantitative methods can complement each other. Cognitive interviews describe how individuals interpret items, and Rasch analysis evaluates measurement properties, which can strengthen the validity of a questionnaire. This approach is encouraged by the development of measurement instruments [71]. Previous psychometric validation studies of the UTAUT have typically relied on classical test theory approaches using structural equation modeling as a preferred method [26]. However, a growing body of literature is applying Rasch analysis for psychometric validation of instruments in health care. This is encouraged in health care science [72] as Rasch models are particularly useful for their ability to assess whether items of a scale fit a single underlying construct, thereby confirming unidimensionality [22]. While psychometric evaluations using Rasch analysis in emergency care, compared with general health care, remain limited [16], this study reflects an early stage of implementation and may therefore influence how constructs are interpreted compared with studies already using technology.

Due to the preliminary implementation stage of video consultations in Swedish EMS, this translated questionnaire contained hypothetical statements. To reduce bias introduced by hypothetical statements, several methodological strategies were used. First, cognitive interviews, as described in the ISPOR guidelines, were conducted with ambulance nurses during the adaptation of the questionnaire, allowing for probing of their understanding of hypothetical items, such as phrases like “in the future,” and refining of wordings for conceptual clarity and to reduce the risk of misinterpretation [29]. This step, combined with research panel review, reduced ambiguities that could have increased hypothetical bias. Second, a pilot study was conducted to test the questionnaire with the possibility for respondents who did not participate in the cognitive interviews to further comment on each item individually. Third, Rasch analysis was used to perform a psychometric evaluation of the questionnaire, assessing measurement properties for prospective intention items on acceptance and use of the technology. The collapsing and reordering of response categories facilitated the psychometric evaluation of the instrument. Although some items lacked data in response categories, this might also indicate that respondents experienced difficulties in distinguishing between response categories. Disordered thresholds are recognized as a psychometric issue when rating scales do not function appropriately [22]. Collapsing response categories is a methodologically appropriate practice to improve scale functioning and ensure that each category provides distinct and ordered information about the latent trait of the construct [73]. This approach has also been reported in other Rasch-based evaluations in health care and is not unique to this study [74]. It is an indication that refinement is warranted when instruments are adapted to a new population or a new context. However, this study cannot determine whether response category disorder reflects the participants’ difficulties in distinguishing between adjacent response categories or the small sample size. This should be investigated further and confirmed with larger samples.

The cognitive interviews and results were interpreted in combination rather than focusing on a single source of analysis (Table 6). This integrated approach strengthened the overall evaluation of the adapted Swedish version questionnaire by enabling the identification of how revisions improved contextual relevance and clarity in relation to participants’ interpretations, while simultaneously revealing psychometric challenges such as disordered thresholds or item misfit. This demonstrates the value of combining qualitative and quantitative methods, as neither approach alone would have identified interpretation and measurement-related issues.

#### Implications for the UTAUT in the Context of Swedish EMS

Our findings indicate that each UTAUT construct (PE, EE, SI, FC, and BI) demonstrated unidimensional properties and are in line with the general concept of the UTAUT, where each construct is a single latent factor [18]. This consistency suggests that conceptual integrity was largely maintained during the translation process into the Swedish prehospital care context. However, the study raises questions about the transferability of a generic translated UTAUT questionnaire, focusing on individual perspective and not taking the specific clinical context into account [63]. Studies using UTAUT in the prehospital context have been published earlier. However, these studies did not provide item-level comparisons [75,76], and there is a significant gap in the literature in prehospital care regarding psychometric evaluations using Rasch analysis on translated versions of prehospital diagnostic or performance scales. Previous UTAUT research within health care identified PE as a main driver for intention to use technology [26] and highlighted organizational and contextual factors for successful implementation [24]. The findings in this study may suggest that determinants relevant for other health care settings may also be relevant for the prehospital context.

This translated questionnaire used hypothetical statements due to the preimplementation stage of video consultation in Swedish EMS. Investigating acceptance of technology relying on hypothetical scenarios, such as asking participants to predict behavior for a system not yet experienced, creates further challenges, such as social desirability bias, and yields a challenge in demonstrating any connection between an attitude and subsequent observed behavior [77]. However, this is a legitimate, valuable, and necessary step for strategic planning before implementation [12], and studies suggest that hypothetical questions can indeed shape future judgment and behavior by increasing the availability of specific knowledge related to the context [78].

Taken together, the UTAUT retained largely structural validity in the context of Swedish EMS, while the interpretation of some constructs appears to shift toward more context-dependent perspectives. This implies that acceptance of technology in EMS cannot be fully understood from an individual perspective alone, but must account for operational conditions, teamwork, and resource availability. Further research is required to refine the UTAUT questionnaire in Swedish EMS and to determine how organizational factors influence individual acceptance of technology.

### Limitations

Some limitations should be considered. This study focused primarily on instrument adaptation, and the limitations relate to sample characteristics and measurement properties. First, the cognitive interview sample may not represent the full diversity of Swedish EMS personnel, and the quantitative psychometric evaluation was constrained by a relatively small sample size and a low response rate of 25.5%. This may introduce selection bias, and the transferability of cognitive interviews and the generalizability of the quantitative findings should therefore be interpreted with caution. Participants for the cognitive interviews were identified by EMS directors, and this may have introduced selection bias. The same individuals participating in both interview rounds may also have influenced their interpretations during the second round. This might have reduced the ability to detect item misfit and increased the risk that problematic items remained undetected during Rasch analysis. Second, the Rasch analysis was based on cross-sectional data from 2 Swedish EMS organizations. The protocol-driven and technology-oriented organizational context of Swedish EMS may have influenced the response pattern, although Rasch models aim to provide sample-independent measurement under model fit assumptions, and may limit the transferability of psychometric findings. Finally, the modification of the original UTAUT response scale categories from 7 to 5 further limits the direct comparison with other UTAUT studies. The need to collapse response categories indicated that the response categories did not function as intended and needed to be adapted. This suggests that a relatively homogeneous sample of participants in the pilot study may have reduced the variability in responses, leading to difficulties distinguishing between adjacent categories. This needs further investigation and further response category refinement. Further cognitive interviews and adaptation with a focus on response categories and a larger sample size for psychometric evaluation of the instrument are needed.

### Conclusions

This study aimed to translate and culturally adapt the UTAUT questionnaire for a Swedish prehospital context and to evaluate its measurement performance at the item level using Rasch analysis. The findings suggest that the translation process resulted in linguistic and contextual relevance, although acceptable psychometric properties were not observed across all constructs in Rasch analysis. There were particular challenges related to response category functioning and the construct of BI. Disordered thresholds indicated that participants had difficulties distinguishing between adjacent response categories, suggesting limited functioning of the rating scale. This indicates that translation and adaptation alone are not sufficient for adequate measurement performance in the context of EMS. Additional refinement and further research with larger samples are warranted before the questionnaire can be recommended for routine and broader use in the Swedish prehospital context.

## Supplementary material

10.2196/96083 Multimedia Appendix 1 Swedish version of the unified theory of acceptance and use of technology questionnaire used in the study.

10.2196/96083 Checklist 1 RULER checklist.

10.2196/96083 Checklist 2 STROBE checklist.

## Abbreviations

ALS: Advanced Life Support
BI: behavioral intention
DIF: differential item functioning
EE: effort expectancy
EMS: emergency medical services
FC: facilitating conditions
HDCI: highest density credible interval
ISPOR: International Society for Pharmacoeconomics and Outcome Research
MSQ: mean square fit statistics
PE: performance expectancy
PSI: person separation index
RULER: Rasch Reporting Guideline for Rehabilitation Research
SI: social influence
STROBE: Strengthening the Reporting of Observational Studies in Epidemiology
TAM: technology acceptance model
UTAUT: unified theory of acceptance and use of technology
WLE-RMU: weighted likelihood estimation relative measurement uncertainty
