# Supplementary material for: Unified Theory of Acceptance and Use of Technology Questionnaire in Swedish Prehospital Care: Translation, Cross-Cultural Adaptation, and Pilot Psychometric Study
Source: JMIR Form Res. 2026 Sep 8;10:e96083. doi: 10.2196/96083 (PMC13552542; doi:10.2196/96083)
Supplement: Supplementary file 1 [file formative-v10-e96083-s002.pdf]

RULER checklist

Recommendation Level	Required	Recommended	Encouraged	Not Encouraged	Reportet In/ At	Note
	**Must appear in the main body of text or supplemental material**	**May be omitted based on context or scope**	**Not required but inclusion follows current "best practices"**	**Does not follow best practices**		
Psychometric domain						
1. Conceptual/content validation methods						
	1. State how content validity was evaluated including the role of the key stakeholders in development of assessment items and final version of the assessment.				p. 8-17	ISPOR guided translation process with ambulance nurses (n=6) involved.
	2. Clearly labelled threshold map or key form may be supplemental material.				p. 6	Multimedia appendix
	3. Figures labelled to describe upper and lower range of both persons and items.				p. 28	Figure 1. Person-item map
	4. Tables of item calibrations ordered hierarchically.				p. 29	Table 5. Item-fit statistics
	5. Rating scale definitions clearly specified.				p. 7-8	5-point Likert scale justified
	6. For new assessments, describe the underlying conceptual model and process of selecting items and rating scale categories definitions.					
	7. For existing assessments, include discussion of prior published hierarchies, when available, and expectations for item order in the current study population.					Not applicable
	8. Discussion section considers how the results of the RM analysis support or challenge the definition of underlying trait.				p. 31-34	Rasch findings linked to discussion
2. Structural validation methods						
2.1. Rating scale structure	1. Specify the type of rating scale model used in the analysis (rating scale, grouped rating scale, and partial credit model).				p. 14	Partial Credit Model
	2. Identify when categories have fewer than 10 responses. Table presenting the number of responses to each rating scale category by item may be supplemental material.				p. 24	
	3. Describe monotonicity of the rating scale categories thresholds.				Multimedia appendix	
	4. Discussion section describes the implications of revising rating scale categories/items (combining score categories) for clinical practice and for interpretation of raw score-to-measure tables.				p. 31-36	
2.2. Unidimensionality; PCAR; item fit; person fit.	5. Report the overall item-trait interaction: overall χ^2, degrees of freedom, and *P* value.	✓Recommend for Winsteps the Smith iteration approach in addition to rule of thumb.<br>✓Recommend first contrast eigenvalue <2; first contrast is <10%.			Not reported	
	6. Provide a table listing fit statistics and statistical significance for each item. State clearly the values for item fit that were used in the study and why these values/ranges were chosen.				p. 15, p. 29	Table 5. Infit MSQ statistics
	7. State clearly the values for PCAR (eigenvalue and percentage variance explained by the first contrast) that were used in the study and why these values/ranges were chosen.				p. 15, 26-33	Eigenvalue <2.0 used as criterion. Table 4.
	8. Discuss reasons why items may have misfit in their particular sample, implications of misfitting items for understanding the underlying trait, and what was done to resolve misfitting items.				p. 31-36	
	9. Demonstrate/discuss the effect (or lack thereof) on person measures whether misfitting items are removed from subsequent iterations of analysis or are retained for conceptual reasons.				Not reported	
	10. Report percentage of misfitting persons.				Not reported	
	11. Discuss reasons why persons in their sample may misfit, implications of misfitting persons for understanding the underlying trait, and what was done to resolve misfitting persons.				Not reported	Not assessed
	12. Demonstrate/discuss misfitting persons who were removed from earlier iterations of analysis are reintroduced after anchoring, their effect on displacement of item calibrations.				Not reported	

Recommendation Level	Required	Recommended	Encouraged	Not Encouraged	Reportet In/ At	Note
	**Must appear in the main body of text or supplemental material**	**May be omitted based on context or scope**	**Not required but inclusion follows current "best practices"**	**Does not follow best practices**		
2.3. Measurement accuracy; PSI, PSR, strata, and internal consistency coefficient (Cronbach α); alignment of items and people	13. Report PSR without persons with extreme person measures. PSI and strata reporting is required for Winsteps users.				p. 30-31	Table 4. PSI reported for all constructs, Crohnbach's alpha as complementary measure
	14. Use Wright's sample independent method (to adjust PSR when data are skewed and/off target).					Not applied
	15. Report floor and ceiling effects when they exceed 15% of the sample.				p. 28	Not formally quantified
	16. Report the mean ± SD of person measures (based on full sample if using repeated measures) and indicate if person ability is greater or less than item challenge.				p. 28	Figure 1.
2.4. Iterations of analysis	17. Include iterations table to facilitate reporting of iterations of analysis (may use supplemental materials).				p. 24	Iterative process described
3. External validation methods						
3.1. Convergent, concurrent, and discriminant validity						
4. Consequential validation methods						
4.1. Sensitivity to change; person level	1. Report cMDC as a supplemental table when the purpose of the tool is to capture person-level changes.				Not applicable	Cross-sectional design
	2. For group-level changes, use Rasch measures to calculate effect sizes. (Note as a limitation for interpreting Rasch measures and discuss as an important topic for future research if it is not appropriate or available to report these in the main psychometric manuscript.)					
4.2. Clinical utility	3. Report the time points at which the items were administered as well as the rehabilitation setting(s).				p. 10-13	EMS setting
	4. Provide the score-to-measure table and describe how clinicians and researchers can interpret and use the key form/threshold map and score-to-measure tables for clinical decision-making.				Not reported	
	5. Comment on the size of the measurement error (standard errors) for interpreting the stability of Rasch measures for individual patients or groups.				p. 27-28	WLE-RMU reported as indicator of measurement uncertainty
	6. Limitation section should include cautionary language when it is logistically challenging to enroll a large group of study participants (eg, a rare disease, or a severe condition such as patients in a coma after a traumatic brain injury) or when it is a pilot study.				p. 38	Small sample size and low response rate
4.3. Societal consequences	7. Report if stakeholders were included in the design or interpretation of the assessment.				p. 10-13	Ambulance nurses
	8. Include future directions for assessment development, such as whether measures are applicable across health care settings and whether the assessment can be applied to all ages, sex and gender identification, ethnicity, and for those with cognitive, hearing, visual, and sensory motor impairments, in the discussion or limitation sections.				p. 35-38	Discussion and limitations sections
5. Measurement invariance, reproducibility, and reliability						
5.1. Measurement invariance; reproducibility; local dependence; repeated measures studies; DIF	1. Report residual item correlations ≥0.2 above the average residual item correlation.				p. 29	Table 4 and multimedia appendix
	2. Demonstrate the consequences of changes on mean person measures and individual person measures using Luppescu's method when revising items.				Not reported	Not applied
	3. State "no consequential local item dependence was found" if no items indicate local item dependence.				p. 26-28	Table 4 and multimedia appendix
	4. State the method used to assess response dependency and discuss the results.				p. 15	Mulitmedia appendix. Assessed using simulation-based cut-offs
	5. For repeated measures, use only 1 time point per person for establishing item calibrations. Comment on the stability of the item hierarchy over time.				Not applicable	Cross-sectional design
	6. Provide clear rationale for choice of categories examined in any DIF analysis. DIF analysis should be relevant to the trait measured in the study and the study population.				Not reported	Only for exploratory purposes. Multimedia appendix
	7. Report on amendments made to the data after detected DIF.					
5.2. Reliability						

Recommendation Level	Required	Recommended	Encouraged	Not Encouraged	Reportet In/ At	Note
	**Must appear in the main body of text or supplemental material**	**May be omitted based on context or scope**	**Not required but inclusion follows current "best practices"**	**Does not follow best practices**		
6. Practical application and clinical implementation						
6.1. Accessibility	1. State in the discussion and/or limitation section if individuals with certain disabilities were excluded from the assessment validation process. 2. Provide a scoring sheet, instruction manual, and other information as supplemental materials; provide a permanent link to this information or state briefly where this information can be obtained regardless of copyright status.				Not reported	No explicit statement
6.2. Training in administration, scoring, and interpretation of the assessment	3. State where users can access the assessment, where they can access the cost of the assessment, and where guidance can be found on administration, scoring, and interpretation.				Not reported	No information provided
6.3. Assessment language	4. State the reading level of the instrument text and seek to use wording and concepts that are inclusive of patients regarding disability status, sex, gender identity, and race and ethnicity when developing or substantially revising assessments.				p. 8-10	Swedish translation and adaptation of UTAUT
