# Supplementary material for: Unified Theory of Acceptance and Use of Technology Questionnaire in Swedish Prehospital Care: Translation, Cross-Cultural Adaptation, and Pilot Psychometric Study
Source: JMIR Form Res. 2026 Sep 8;10:e96083. doi: 10.2196/96083 (PMC13552542; doi:10.2196/96083)
Supplement: Supplementary file 3 [file formative-v10-e96083-s001.docx]

Multimedia appendix 1

**Unified Theory of Acceptance and Use of Technology questionnaire in Swedish Prehospital Care: Translation, Cross-Cultural Adaptation, and Psychometric Study**

Contents

[Cognitive interview according to ISPOR	2](#_Toc225153220)

[**Figure 1**. Permission from the original author to translate the original version into Swedish	2](#_Toc225153221)

[**Table 1**. Basic information about the translators	2](#_Toc225153222)

[**Table 2.** Information of the research panel	3](#_Toc225153223)

[**Table 3.** Basic information about participants	4](#_Toc225153224)

[**Table 4.** The original version, forward translation version, reconciliation, back translation and versions of harmonization	5](#_Toc225153225)

[**Table 5.** The translated version of response categories from in collaboration with the professional translator	13](#_Toc225153226)

[**Figure 2.** Second Email conversation with the author with a specific question about hypothetical item statements, December 9^th^, 2026	14](#_Toc225153227)

[**Table 6.** Revised UTAUT questionnaire based on the feedback of respondents	14](#_Toc225153228)

[**Table 7**. Response categories for the pilot study	18](#_Toc225153229)

[**Table 8**. Final version after two rounds of cognitive debriefing	18](#_Toc225153230)

[**Rasch analysis**	20](#_Toc225153231)

[**Performance Expectancy**	20](#_Toc225153232)

[**Social Influence**	26](#_Toc225153233)

[**Facilitating Conditions**	29](#_Toc225153234)

[**Behavioral Intention**	32](#_Toc225153235)

# Cognitive Interviews According to ISPOR

## **Figure 1**. Permission from the original author to translate the original version into Swedish

**Table 1**. Basic information about the translators involved in the forward translation, reconciliation, back translation, back translation review and harmonisation

| **Researcher/**  **participant** | **Educational background** | **Field of research** | **Awareness of the** | **Translation task** |
| --- | --- | --- | --- | --- |
| 1 | Postgraduate degree | Out-of-hospital Cardia Arrest, Prehospital care, Anaesthesia | Yes | Forward translation  Reconciliation  Backward translation review  Harmonisation |
| 2 | Postgraduate student | None | Yes | Forward translation Reconciliation  Backward translation review  Harmonisation |
| 3 | Authorised professional translator | None | No | Back translation  Back translation review  Harmonisation |

**Table 2.** Information of the research panel contributing to the translation and adaptation of UTAUT

| **Contributor** | **Educational background** | **Function** | **Contribution** | **Field of research** |
| --- | --- | --- | --- | --- |
| 1 | Postgraduate degree | Main supervisor | Conceptualization, Methodology, forward translation, reconciliation, back translation, back translation review, harmonization, review of cognitive debriefing results and finalization, proofreading, reviewing final report. | Out-of-hospital Cardiac Arrest, Prehospital care, Anaesthesia. |
| 2 | Master’s degree | Doctoral student | Conceptualization, Preparation, forward translation, Reconciliation, back translation, back translation review, harmonization, cognitive debriefing, review of cognitive debriefing results and finalization, proofreading and final report. | Prehospital care education, clinical nursing in prehospital care |
| 3 | Postgraduate degree | Co-author | Methodological consulting, review of items prior to and after cognitive debriefings. | Measurement methodology and psychometrics |
| 4 | Postgraduate degree | Co-supervisor | Methodology, review of items prior to and after cognitive debriefings. | Prehospital Care |
| 5 | Postgraduate degree | Co-supervisor | Methodology, review of items prior to and after cognitive debriefings. | Pain and rehabilitation  Biomechanics |

**Table 3.** Basic information about participants for both cognitive interviews

| **First and second round of cognitive interview** | | | | | |
| --- | --- | --- | --- | --- | --- |
| **Participant ID** | **Ambulance region** | Age | Sex | **Time for interview 1** | **Time for interview 2** |
| **1** | 1 | 52 | M | 01:21 | 00:51 |
| **2** | 2 | 63 | M | 00:45 | 00:31 |
| **3** | 1 | 55 | M | 00:52 | 00:26 |
| **4** | 2 | 35 | M | 00:51 | 00:35 |
| **5** | 1 | 34 | F | 00:45 | 00:38 |
| **6** | 2 | 43 | M | 00:36 | 00:30 |

| **Table 4.** The original version, forward translation version, reconciliation, back translation and versions of harmonization | | | | | | | | |
| --- | --- | --- | --- | --- | --- | --- | --- | --- |
| **Original version** | **FT1** | **FT2** | **Reconciliation** | **BT** | **BT review** | **H1** | **H2** | **H3** |
| **Performance expectancy** | Förväntad prestation | Förväntad prestation | Genomförande | Implementation | Implementation  *Titeln för den här item kommer att bytas tillbaka till originaltiteln Performance expectancy och på svenska blir det ”Förväntad nytta”* | Performance expectancy | Förväntad nytta | Förväntad nytta |
| U6: I would find the system useful in my job. | Jag anser att videokonsultation i ambulanssjukvården skulle vara användbart i mitt arbete. | Jag skulle tycka att systemet är användbart i mitt jobb. | Jag anser att digital teknik som exempelvis videokonsultation är användbart i mitt jobb.  *Bestämde oss för ”anser”* | I believe that digital technology, such as video consultation, is useful in my job. | I believe that digital technology, such as video consultation, is useful in my job.  *Detta item är ok.* | I believe that digital technology, such as video consultation, is useful in my job. | Jag anser att digital teknik som exempelvis videokonsultation är användbart i mitt jobb. | Jag anser att den digitala tekniken (exempelvis videokonsultation) är användbar i mitt jobb. |
| RA1: Using the system enables me to accomplish tasks more quickly. | Användningen av videokonsultation gör att jag kan utföra uppgifter snabbare. | Att använda systemet gör att jag kan utföra uppgifter snabbare. | Att använda digital teknik som exempelvis videokonsultation, gör att jag kan utföra uppgifter snabbare.  *Item är ok.* | Using digital technology, such as video consultation, means that I can execute tasks more quickly. | Using digital technology, such as video consultation, means that I can execute tasks more quickly.  *Dettaa item är ok.* | Using digital technology, such as video consultation, means that I can execute tasks more quickly. | Att använda digital teknik som exempelvis videokonsultation, gör att jag kan utföra uppgifter snabbare. | Att använda digital teknik (exempelvis videokonsultation) gör att jag kan utföra uppgifter snabbare. |
| RA5: Using the system increases my productivity | Användningen av videokonsultation i ambulanssjukvården skulle öka min effektivitet. | Att använda systemet ökar min produktivitet. | Att använda digital teknik som exempelvis videokonsultation, ökar min produktivitet.  *Använder engelska ordet till svensk version* | Using digital technology, such as video consultation, increases my productivity. | Using digital technology, such as video consultation, increases my productivity.  *Detta item är ok.* | Using digital technology, such as video consultation, increases my productivity. | Att använda digital teknik som exempelvis videokonsultation, ökar min produktivitet. | Genom att använda digital teknik (exempelvis videokonsultation) ökar min produktivitet. |
|  |  |  |  |  |  |  |  |  |
|  |  |  |  |  |  |  |  |  |
|  |  |  |  |  |  |  |  |  |
|  |  |  |  |  |  |  |  |  |
|  |  |  |  |  |  |  |  |  |
|  |  |  |  |  |  |  |  |  |
|  |  |  |  |  |  |  |  |  |
| **Original version** | **FT1** | **FT2** | **Reconciliation** | **BT** | **BT review** | **H1** | **H2** | **H3** |
| OE7: If I use the system, I will increase my chances of getting a personal expectations raise.  OE1: If I use the system, increase my effectiveness on the job.  OE3: If I use the system, I will increase the quality of output of my job. | Användningen av videokonsultation skulle förändra kvalitén i den vård som ges till patienten. | Om jag använder systemet ökar jag mina chanser att bli bekräftad (OE7). | Användningen av digital teknik som exempelvis videokonsultation skulle öka kvalitén i den vård som ges till patienten.  *Här byter vi ut ett item OE7 används inte för den är inte relevant i kontexten. OE3 används istället som fokuserar på kvalitén av jobbet som utförs.* | Use of digital technology, such as video consultation, would increase the quality of the care provided to the patient. | Use of digital technology, such as video consultation, would increase the quality of the care provided to the patient.  *Detta item kan vara för kontextuellt, men vi lämnar det så fram tills vi har gått igenom kognitiv debriefing.* | Use of digital technology, such as video consultation, would increase the quality of the care provided to the patient. | Användningen av digital teknik som exempelvis videokonsultation skulle öka kvalitén i den vård som ges till patienten. | Användning av digital teknik (exempelvis videokonsultation) ökar kvalitén i den vård som ges till patienten. |
|  |  |  |  |  |  |  |  |  |
|  |  |  |  |  |  |  |  |  |
|  |  |  |  |  |  |  |  |  |
|  |  |  |  |  |  |  |  |  |
|  |  |  |  |  |  |  |  |  |
|  |  |  |  |  |  |  |  |  |
|  |  |  |  |  |  |  |  |  |
|  |  |  |  |  |  |  |  |  |
|  |  |  |  |  |  |  |  |  |
|  |  |  |  |  |  |  |  |  |
|  |  |  |  |  |  |  |  |  |
|  |  |  |  |  |  |  |  |  |
|  |  |  |  |  |  |  |  |  |
|  |  |  |  |  |  |  |  |  |
|  |  |  |  |  |  |  |  |  |
| **Original version** | **FT1** | **FT2** | **Reconciliation** | **BT** | **BT review** | **H1** | **H2** | **H3** |
| **Effort expectancy** | Förväntad ansträngning | Förväntad andsträngning | Enkelhet  *Vi vände om ordet från ett negative till en mer positiv ton.* | Simplicity | Simplicity *Här har vi enats om att titeln kommer at heta ”Användarvänlighet” och även här är titeln tillbaka till den ursprungliga versionen ”Effort expectancy”* | Effort expectancy | Användarvänlighet | Användarvänlighet |
| EOU3: My interaction with the system would be clear and understandable. | Min interaktion med systemet för videokonferens skulle vara klart och tydligt. EOU3 | Mitt samspel med systemet ska vara tydligt och förståeligt. | Mitt deltagande i samband med digital teknik som exempelvis videokonsultation, bör vara tydligt och förståeligt.  *Ordet ”interaction” gav en del diskussioner. Men vi landade i att använda deltagandet för att komma vidare.* | My participation in connection with digital technology, such as video consultation, should be clear and comprehensible. | My participation in connection with digital technology, such as video consultation, should be clear and comprehensible.  *Vi byter ut “should be” till “would be” för att det antas att videokonsultation är redan igång.* | My participation in connection with digital technology, such as video consultation, would be clear and comprehensible. | Mitt deltagande i samband med digital teknik som exempelvis videokonsultation, bör vara tydligt och förståeligt. Här ändrade vi från ”should be” till ”would be”. Ordet ”Bör" motsvarar "should" på engelska, vilket uttrycker en rekommendation eller förväntan. "Skulle" motsvarar "would" och kan uppfattas som mer hypotetiskt men med en mindre bestämd ton.Ska vi byta till ”skulle” eller ska vi avvakta kognitiv debriefing? | Mitt engagemang som rör digital teknik (exempelvis videokonsultation) bör vara tydligt och förståeligt. |
|  |  |  |  |  |  |  |  |  |
|  |  |  |  |  |  |  |  |  |
|  |  |  |  |  |  |  |  |  |
|  |  |  |  |  |  |  |  |  |
|  |  |  |  |  |  |  |  |  |
| **Original version** | **FT1** | **FT2** | **Reconciliation** | **BT** | **BT review** | **H1** | **H2** | **H3** |
| EOU5: It would be easy for me to become skillful at using the system | Det skulle vara lätt för mig att bli skicklig på att använda videokonsultation. | Det skulle vara lätt för mig att bli skicklig på att använda systemet. | Det bör vara lätt för mig att bli skicklig i använda digital teknik som exempelvis videokonsultation.  *Item är ok* | It should be easy for me to become proficient in using digital technology, such as video consultation. | It should be easy for me to become proficient in using digital technology, such as video consultation.  *Vi byter ut “should be” till “would be” för att det antas att videokonsultation är redan igång.* | It would be easy for me to become proficient in using digital technology, such as video consultation. | Det bör vara lätt för mig att bli skicklig i använda digital teknik som exempelvis videokonsultation.  Se ovan, gäller *bör* | Det bör vara lätt att bli en skicklig användare inom digital teknik (exempelvis videokonsultation). |
| EOU6: I would find the system easy to use. | Jag skulle tycka att systemet för videokonsultation är lätt att använda. EOU6 | Jag skulle tycka att systemet är lätt att använda. | Den digitala tekniken som exempelvis videokonsultation, bör vara lätt att använda.  *Item är ok, anpassad till kontexten.* | The digital technology, such as video consultation, should be easy to use. | The digital technology, such as video consultation, should be easy to use.  *Vi byter ut “should be” till “would be” för att det antas att videokonsultation är redan igång.* | The digital technology, such as video consultation, would be easy to use. | Den digitala tekniken som exempelvis videokonsultation, bör vara lätt att använda.  Se ovan, gäller bör. | Digital teknik (exempelvis videokonsultation) bör vara lätt att använda. |
| EU4: Learning to operate the system is easy for me. | Att lära mig använda videokonsultation skulle vara lätt för mig. EU4. | Att lära sig använda systemet är lätt för mig. | Att lära sig att använda digital teknik som exempelvis videokonsultation, är lätt för mig. *Item är ok.* | Learning to use digital technology, such as video consultation, is easy for me. | Learning to use digital technology, such as video consultation, is easy for me.  *Detta item är ok.* | Learning to use digital technology, such as video consultation, is easy for me. | Att lära sig att använda digital teknik som exempelvis videokonsultation, är lätt för mig. | Att lära sig att använda digital teknik (exempelvis videokonsultation) är lätt för mig. |
|  |  |  |  |  |  |  |  |  |
|  |  |  |  |  |  |  |  |  |
|  |  |  |  |  |  |  |  |  |
| **Original version** | **FT1** | **FT2** | **Reconciliation** | **BT** | **BT review** | **H1** | **H2** | **H3** |
| **Social influence** | Socialt inflytande | Social inflytande | Socialt inflytande  *Ok* | Social influence | Social influence  *Denna titel kommer att ändras till ”påverkan” på svenska. För att inflytande som är den tekniska ordagranna översättningen tyder på att inflytande är ett medveten handling riktad mot en person med syftet att ändra ett beteende.* | Social influence | Social påverkan | Social påverkan |
| SN1: People who influence my behavior think that I should use the system. | Personer som påverkar mitt beteende anser att jag borde använda videokonsultation. SN1. | Folk som påverkar mitt beteende och tycker att jag ska använda systemet. | Personer som påverkar mitt beteende tycker att jag borde använda systemet. *Enar oss om ordet”personer”.* | People who influence my behaviour think I should use the system. | People who influence my behaviour think I should use the system.  *Detta item är ok.* | People who influence my behaviour think I should use the system. | Personer som påverkar mitt beteende tycker att jag borde använda systemet. | Jag påverkas av andra till att använda system. |
| SN2: People who are important to me think that I should use the system. | Personer om är viktiga för mig anser att jag borde använda videokonsultation. SN2 | Människor som är viktiga för mig och tycker att jag ska använda systemet. | Personer som är viktiga för mig tycker att jag borde använda digital teknik som exempelvis videokonsultation.  *Enar oss om ordet”personer”* | People who are important to me think I should use digital technology, such as video consultation. | People who are important to me think I should use digital technology, such as video consultation.  *Detta item är ok.* | People who are important to me think I should use digital technology, such as video consultation. | Personer som är viktiga för mig tycker att jag borde använda digital teknik som exempelvis videokonsultation. | Personer som är viktiga runt omkring mig tycker jag bör använda digital teknik (exempelvis videokonsultation). |
|  |  |  |  |  |  |  |  |  |
|  |  |  |  |  |  |  |  |  |
|  |  |  |  |  |  |  |  |  |
| **Original version** | **FT1** | **FT2** | **Reconciliation** | **BT** | **BT review** | **H1** | **H2** | **H3** |
| SF2: The senior management of this business has been helpful in the use of the system. | Jag tror att verksamhetsledningen skulle vara behjälplig vid användning av videokonsultation. SF2. | Ledningen för ambulansverksamheten har varit till hjälp i användningen av systemet. | Verksamhetsledningen har varit behjälplig i användningen av digital teknik som exempelvis videokonsultation.  *”Verksamhetsledning” är ett enklare ord.* | The operative management has provided assistance in the use of digital technology, such as video consultation. | The operative management has provided assistance in the use of digital technology, such as video consultation.  *Här byter vi ut operative management mot senior management med tanke på en generaliserbarhet och att det är beslutsfattarna som avses med detta. På svenska kommer ”verksamhetsledningen” användas.* | The senior management has provided assistance in the use of digital technology, such as video consultation. | Verksamhetsledningen har varit behjälplig i användningen av digital teknik som exempelvis videokonsultation. | Verksamhetsledningen är behjälplig i användning av digital teknik (exempelvis videokonsultation). |
| SF4: In general, the organization has supported the use of the system. | Ambulanssjukvården kommer att ge mig stöd i att använda videokonsultation. | Generellt sett har organisationen supportat användningen av systemet. | Överlag så stöttar organisationen användning av digital teknik. *Byter ”Generellt” till ”överlag” som är mer tilltalande* | In general, the organisation supports use of digital technology. | In general, the organisation supports use of digital technology.  *Detta item är ok.* | In general, the organisation supports use of digital technology. | Överlag så stöttar organisationen användning av digital teknik. | Överlag så stöttar organisationen användning av digital teknik. |
| **Facilitating conditions** | Underlättande faktorer | Faktorer som underlättar | Underlättande omständigheter *Väljer omständigheter istället för faktor. Enklare att förstå.* | Facilitating cirumstances | Facilitating circumstances *Denna titel kommer att ändras till ”Facilitating conditions” som på engelska, då originalversionen är mer precis. På svenska kommer titeln heta ”Omständigheter som underlättar”.* | Facilitating conditions | Omständigheter som underlättar | Omständigheter som underlättar |
|  |  |  |  |  |  |  |  |  |
|  |  |  |  |  |  |  |  |  |
|  |  |  |  |  |  |  |  |  |
|  |  |  |  |  |  |  |  |  |
| **Original version** | **FT1** | **FT2** | **Reconsiliation** | **BT** | **BT review** | **H1** | **H2** | **H3** |
| PBC2: I have the resources necessary to use the system. | Jag har resurser som krävs för att använda videokonsultation. | Jag har de resurser som krävs för att använda systemet. | Jag har de resurser som krävs för att använda digital teknik som exempelvis videokonsultation.  *Item är ok* | I have the resources required to use digital technology, such as video consultation. | I have the resources required to use digital technology, such as video consultation. *Här kommer vi att byta ut ordet ”required” mot ” neccesary” som i originalversionen. Anledningen är att required är mer ett krav att ha en förmåga och ”neccesary” är mer ett behov.* | I have the resources necessary to use digital technology, such as video consultation. | Jag har de resurser som krävs för att använda digital teknik som exempelvis videokonsultation. | Jag har resurser som krävs för att använda digital teknik (exempelvis videokonsultation). |
| PBC3: I have the knowledge necessary to use the system. | Jag har den kunskap som krävs för att använda videokonsultation. | Jag har den kunskap som krävs för att använda systemet. | Jag har den kunskap som krävs för att använda digital teknik som exempelvis videokonsultation.  *Item är ok* | I have the knowledge required to use digital technology, such as video consultation. | I have the knowledge required to use digital technology, such as video consultation.. *Här kommer vi att byta ut ordet ”required” mot ” necessary” som i originalversionen. Anledningen är att required är mer ett krav att ha en förmåga och ”neccesary” är mer ett behov.* | I have the knowledge necessary to use digital technology, such as video consultation. | Jag har den kunskap som krävs för att använda digital teknik som exempelvis videokonsultation. | Jag har den kunskap som krävs för att använda digital teknik (exempelvis videokonsultation). |
| PBC5: The system is not compatible with other systems I use. | Videokonsultation är inte kompatibelt med andra system som jag använder. | Systemet är inte kompatibelt med andra system jag använder. | Digital teknik som exempelvis videokonsultation är inte kompatibelt med andra system som jag använder. *Item är ok* | Digital technology, such as video consultation, is not compatible with other systems that I use. | Digital technology, such as video consultation, is not compatible with other systems that I use.  *Detta item är ok* | Digital technology, such as video consultation, is not compatible with other systems that I use. | Digital teknik som exempelvis videokonsultation är inte kompatibelt med andra system som jag använder. | Digital teknik (exempelvis videokonsultation) är inte kompatibel med andra system som jag använder. |
| FC3: A specific person (or group) is available for assistance with system | En specifik person skulle vara tillgänglig för att bistå med systemet för videokonferens. FC3 | En specifik person/support som är tillgänglig om problem uppstår med systemet. | En specifik person finns tillgänglig för att bistå vid systemproblematik.  *Item är ok* | A specific person is available to assist in case of system problems. | A specific person is available to assist in case of system problems.  *Detta item är ok* | A specific person is available to assist in case of system problems. | En specifik person finns tillgänglig för att bistå vid systemproblematik. | En specifik person finns tillgänglig för att bistå vid systemproblematik. |
|  |  |  |  |  |  |  |  |  |
| **Original version** | **FT1** | **FT2** | **Reconciliation** | **BT** | **BT review** | **H1** | **H2** | **H3** |
| **Behavioral intention** | Beteendeintention att använda systemet | Beteendemässig avsikt att använda systemet | Beteendemässig avsikt att använda systemet | Behavioural intent to use the system | Behavioural intent to use the system *Denna titel är ok* | Behavioural intent to use the system | Beteendemässig avsikt att använda systemet | Beteendemässig avsikt att använda systemet |
| BI1: I intend to use the system in the next <n> months | Jag avser att använda videokonsultation under de närmaste 12 månaderna. | Jag tänker använda systemet under de kommande <n> månaderna. | Jag avser att använda den digitala tekniken under de kommande 12 månaderna.  *Item är ok Item är ok, vi bestämde oss initial att använda en kategorisk siffra.* | I intend to use the digital technology during the next 12 months. | I intend to use the digital technology during the next 12 months.  *Detta item är ok, på svenska kommer vi inte att ha en tidsangivelse utan vi kommer att skriva på sikt. Här förväntar vi oss klarhet efter kognitiva intervjuer för vilken term vi ska använda.* | I intend to use the digital technology during the next 12 months. | Jag avser på sikt att använda den digitala tekniken som exempelvis videokonsultation.*Här bestämde vi oss att använda av ett ospecifik tidsram för att vi inte vet när videokonsultation kommer att införas. Orsaken till ändringen är kontextberoende.* | Jag avser på sikt att använda digital teknik (exempelvis videokonsultation). |
| B12: I predict I would use the system in the next <n> months | Jag förutser att jag kommer att använda videokonsultation under de närmaste 12 månaderna | Jag förutspår att jag skulle använda systemet under de kommande <n> månaderna. | Jag förutser att jag kommer att använda digital teknik som exempelvis videokonsultation under de kommande 12 månaderna.  *Item är ok, vi bestämde oss initial att använda en kategorisk siffra* | I predict that I will use digital technology, such as video consultation, during the next 12 months. | I predict that I will use digital technology, such as video consultation, during the next 12 months.  *Detta item är ok, på svenska kommer vi inte att ha en tidsangivelse utan vi kommer att skriva på sikt. Här förväntar vi oss klarhet efter kognitiva intervjuer för vilken term vi ska använda.* | I predict that I will use digital technology, such as video consultation, during the next 12 months. | Jag förutser på sikt att jag kommer att använda digital teknik som exempelvis videokonsultation. *Här bestämde vi oss att använda av ett ospecifik tidsram för att vi inte vet när videokonsultation kommer att införas. Orsaken till ändringen är kontextberoende* | Jag förutser att jag på sikt kommer att använda digital teknik (exempelvis videokonsultation). |
|  |  |  |  |  |  |  |  |  |
|  |  |  |  |  |  |  |  |  |
| **Original version** | **FT1** | **FT2** | **Reconsiliation** | **BT** | **BT review** | **H1** | **H2** | **H3** |
| B13: I plan to use the system in the next <n> months. | Jag planerar att använda videokonsultation under de kommande 12 månaderna. | Jag planerar att använda systemet under de kommande <n> månaderna. | Jag planerar att använda digital teknik som exempelvis videokonsultation under de kommande 12 månader.  *Item är ok, vi bestämde oss initial att använda en kategorisk siffra.* | I plan to use digital technology, such as video consultation, during the next 12 months. | I plan to use digital technology, such as video consultation, during the next 12 months.  *Detta item är ok, på svenska kommer vi inte att ha en tidsangivelse utan vi kommer att skriva på sikt. Här förväntar vi oss klarhet efter kognitiva intervjuer för vilken term vi ska använda.* | I plan to use digital technology, such as video consultation, during the next 12 months. | Jag planerar på sikt att använda digital teknik som exempelvis videokonsultation.  *Här bestämde vi oss att använda av ett ospecifik tidsram för att vi inte vet när videokonsultation kommer att införas. Orsaken till ändringen är kontextberoende* | På sikt planerar jag att använda mig av digital teknik (exempelvis videokonsultation). |

The table summarizes the original version, two forward translation versions, one reconciliation version, and two back translation versions of which one is the back translation review and three harmonization version showing the iterative process of the UTAUT questionnaire before round 1 of cognitive interviews.

FT1: Forward translation contributor 2, FT2: Forward translation contributor 2, BT: Back translation, BT review: Back translation review, H1: back translation English version, H2: Harmonization round with research panel, H3: Grammatical adjustment to improve readability and flow.

## **Table 5.** The translated version of response categories from in collaboration with the professional translator

| 1 = do not agree at all | 2 = do not agree entirely | 3 = neither agree nor disagree | 4 = partially agree | 5 = strongly agree |
| --- | --- | --- | --- | --- |
|  |  |  |  |  |

**Figure 2.** Second Email conversation with the author with a specific question about hypothetical item statements, December 9^th^, 2026

## **Table 6.** Revised UTAUT questionnaire based on the feedback of respondents

| **Questionnaire used for the first round of cognitive debriefing interviews. December 2024** | **Revised questionnaire after expert panel meeting and submitted to respondents for a second round of cognitive debriefing interviews. December 2024-January 2025** | **Revised questionnaire after reconciliation with expert panel and adjustments based on the second round of cognitive interviews. 2025-02-06 and 2025-02-10** |
| --- | --- | --- |
| **Förväntad nytta** | **Förväntad nytta** | **Förväntad nytta** |
| Jag anser att den digitala tekniken (exempelvis videokonsultation) är användbar i mitt jobb. | Videokonsultation kommer att vara användbart i mitt arbete. | Videokonsultation kommer att vara användbart i mitt arbete. |
| Att använda digital teknik (exempelvis videokonsultation) gör att jag kan utföra uppgifter snabbare. | Användning av videokonsultation kommer att göra att jag kan utföra arbetsuppgifter snabbare. | Användning av videokonsultation kommer att göra att jag kan utföra arbetsuppgifter snabbare inom ambulanssjukvården. |
| Genom att använda digital teknik (exempelvis videokonsultation) ökar min produktivitet.  *Respondent were confused about the term “productivity* | Videokonsultation kommer att göra att jag kan påverka ambulanssjukvårdens tillgänglighet.  Based on the feedbacks we defined productivity as available ambulance resources | Videokonsultation kommer att bidra till en ökad tillgänglighet för nya ambulansuppdrag. |
|  |  |  |
|  |  |  |
| **Questionnaire used for the first round of cognitive debriefing interviews. December 2024** | **Revised questionnaire after expert panel meeting and submitted to respondents for a second round of cognitive debriefing interviews. December 2024-January 2025** | **Revised questionnaire after reconciliation with expert panel and adjustments based on the second round of cognitive interviews. 2025-02-06 and 2025-02-10** |
|  |  |  |
| Användning av digital teknik (exempelvis videokonsultation) ökar kvalitén i den vård som ges till patienten. | Användning av videokonsultation kommer att öka kvalitén i den vård som ges till patienten. | Användning av videokonsultation kommer att öka kvalitén i den vård som ges till patienten. |
| **Användarvänlighet** | **Användarvänlighet** | **Användervänlighet** |
| Mitt engagemang som rör digital teknik (exempelvis videokonsultation) bör vara tydligt och förståeligt.  *The statement was unclear to the respondents. The item had to be explained during the interviews.* | Det kommer att vara tydligt och lättförståeligt hur jag ska använda videokonsultation. | Det kommer att vara tydligt och lättförståeligt hur jag ska använda videokonsultation |
| Det bör vara lätt att bli en skicklig användare inom digital teknik (exempelvis videokonsultation). | Det kommer att vara lätt för mig som ambulanspersonal att bli en skicklig användare av videokonsultation. | Det kommer att vara lätt för mig att bli en skicklig användare av videokonsultation. |
| Digital teknik (exempelvis videokonsultation) bör vara lätt att använda. | Videokonsultation kommer att vara användarvänligt | Jag förväntar mig att videokonsultation kommer att vara användarvänligt. |
| Att lära sig att använda digital teknik (exempelvis videokonsultation) är lätt för mig. | Videokonsultation kommer att vara lätt för mig att lära sig | Videokonsultation kommer att vara lätt för mig att lära mig. |
| **Social påverkan** | **Social påverkan** | **Social påverkan** |
| Jag påverkas av andra till att använda system.  *Item was unclear. The term “system” was used here, all other items used the word “ digital teknik (exempelvis videokonsultation).* | Personer jag samarbetar med, kommer influera mig att använda videokonsultation. | Personer jag samarbetar med kommer influera mig att använda videokonsultation. |
| Personer som är viktiga runt omkring mig tycker jag bör använda digital teknik (exempelvis videokonsultation). | Personer jag samarbetar med, kommer att tycka att jag ska använda videokonsultation. | Personer jag samarbetar med, kommer att tycka att jag ska använda videokonsultation. |
| Verksamhetsledningen är behjälplig i användning av digital teknik (exempelvis videokonsultation).  *The term ”behjälplig” was percieved as confusing and could be interpreted differntly depending on the context.* | Verksamhetsledningen kommer att vara behjälplig i att använda videokonsultation. | Verksamhetsledningen kommer att vara behjälplig när jag börjar använda videokonsultation. |
|  |  |  |
| **Questionnaire used for the first round of cognitive debriefing interviews. December 2024** | **Revised questionnaire after expert panel meeting and submitted to respondents for a second round of cognitive debriefing interviews. December 2024-January 2025** | **Revised questionnaire after reconciliation with expert panel and adjustments based on the second round of cognitive interviews. 2025-02-06 and 2025-02-10** |
| Överlag så stöttar organisationen användning av digital teknik. | I sin helhet kommer ambulanssjukvården stötta användning av videokonsultation. | I sin helhet kommer ambulanssjukvården stötta användning av videokonsultation. |
| **Omständigheter som underlättar** | **Omständigheter som underlättar** | **Omständigheter som underlättar** |
| Jag har resurser som krävs för att använda digital teknik (exempelvis videokonsultation).  *Respondents had difficulties to understand the meaning of the word “resurser” and this word could be interpreted differently depending on the context. They suggested a revision to be more specific.* | Jag kommer att ha förutsättningar som krävs för att använda videokonsultation. | Jag kommer att ha de förutsättningar som krävs för att använda videokonsultation. |
| Jag har den kunskap som krävs för att använda digital teknik (exempelvis videokonsultation). | Jag kommer att ha den kunskap som krävs för att använda videokonsultation. | Jag kommer att ha den kunskap som krävs för att använda videokonsultation. |
| Digital teknik (exempelvis videokonsultation) är inte kompatibel med andra system som jag använder.  *The word “digital teknik” should be replaced by “videokonsultation”. Also, this item has a negative word “inte” and to reduce ambiguities and double negation, this item should be revised.* | Videokonsultation kommer att vara kompatibel med andra digitala system som jag använder. | Videokonsultation kommer att vara kompatibel med andra digitala system som jag använder. |
| En specifik person finns tillgänglig för att bistå vid systemproblematik.  *As mentioned above, the word “digital teknologi” should be replaced even in this item.* | Det kommer att finnas teknisk support tillgänglig för att kunna bistå om tekniska problem uppstår i samband med videokonsultation. | Det kommer alltid att finnas teknisk support tillgänglig vid behov |
|  |  |  |
|  |  |  |
|  |  |  |
|  |  |  |
|  |  |  |
|  |  |  |
|  |  |  |
|  |  |  |
|  |  |  |
|  |  |  |
|  |  |  |
|  |  |  |
|  |  |  |
| **Questionnaire used for the first round of cognitive debriefing interviews. December 2024** | **Revised questionnaire after expert panel meeting and submitted to respondents for a second round of cognitive debriefing interviews. December 2024-January 2025** | **Revised questionnaire after reconciliation with expert panel and adjustments based on the second round of cognitive interviews. 2025-02-06 and 2025-02-10** |
| **Beteendemässig avsikt att använda systemet** | **Beteendemässig avsikt att använda systemet** | **Beteendemässig avsikt att använda systemet** |
| Jag avser på sikt att använda digital teknik (exempelvis videokonsultation).  *The meaning ”på sikt” is too ambiguous and should be replaced by an number or time frame.* | Under förutsättning att verksamheten implementerar videokonsultation så avser jag att använda det inom ett år.  *Based on expert panel suggestion, it was important to inform respondents about the willingness of the organisation to introduce video consultation. This reliefs the cognitive load of respondents to question, whether the organisation is willing or not to implement video consultations. Hence a contextual adaptation of the item.* | Under förutsättning att verksamheten implementerar videokonsultation, avser jag att använda det inom ett år. |
| Jag förutser att jag på sikt kommer att använda digital teknik (exempelvis videokonsultation). | Under förutsättning att verksamheten implementerar videokonsultation så förutser jag att använda det inom ett år. | Under förutsättning att verksamheten implementerar videokonsultation, kommer jag att använda det så snart det är tillgängligt.  *Major revision, see Final Report rationale.* |
| På sikt planerar jag att använda mig av digital teknik (exempelvis videokonsultation).  *Even here, the term “på sikt” is not clear enough and revision is to be considered* | Under förutsättning att verksamheten implementerar videokonsultation så planerar jag att använda det inom ett år. | Jag planerar att aktivt använda videokonsultation i mitt dagliga arbete efter det har implementerats i verksamheten.  *Major revision, see Final report rationale* |

**Table 7**. Response categories for the pilot study based on consensus in the research group

The initial response categories which were decided to use for the pilot study were as follows:

| **Swedish version** | **English translation** |
| --- | --- |
| Instämmer inte alls  Instämmer delvis inte  Varken instämmer eller instämmer inte  Instämmer delvis  Instämmer helt | Strongly disagree  Somewhat disagree / Partly disagree  Neither agree nor disagree  Somewhat agree / Partly agree  Strongly agree |

**Table 8**. Final version after two rounds of cognitive debriefing interview and research panel reviews

**Final version after two rounds of cognitive debriefing interview and research panel reviews**

Item 1

**Videokonsultation kommer att vara användbart i mitt arbete**

Item 2

**Användning av videokonsultation kommer att göra att jag kan utföra arbetsuppgifter snabbare inom ambulanssjukvården.**

Item 3

**Videokonsultation kommer att bidra till en ökad tillgänglighet för nya ambulansuppdrag.**

Item 4

**Användning av videokonsultation kommer att öka kvalitén i den vård som ges till patienten.**

Item 5

**Det kommer att vara tydligt och lättförståeligt hur jag ska använda videokonsultation**

Item 6

**Det kommer att vara lätt för mig att bli en skicklig användare av videokonsultation.**

Item 7

**Jag förväntar mig att videokonsultation kommer att vara användarvänligt.**

Item 8

**Videokonsultation kommer att vara lätt för mig att lära mig.**

Item 9

**Personer jag samarbetar med kommer influera mig att använda videokonsultation.**

Item 10

**Personer jag samarbetar med, kommer att tycka att jag ska använda videokonsultation.**

Item 11

**Verksamhetsledningen kommer att vara behjälplig när jag börjar använda videokonsultation.**

Item 12

**I sin helhet kommer ambulanssjukvården stötta användning av videokonsultation.**

Item 13

**Jag kommer att ha de förutsättningar som krävs för att använda videokonsultation**

Item 14

**Jag kommer att ha den kunskap som krävs för att använda videokonsultation**

Item 15

**Videokonsultation kommer att vara kompatibel med andra digitala system som jag använder**

Item 16

**Det kommer alltid att finnas teknisk support tillgänglig vid behov**

Item 17

**Under förutsättning att verksamheten implementerar videokonsultation, avser jag att använda det inom ett år.**

Item 18

**Under förutsättning att verksamheten implementerar videokonsultation, kommer jag att använda det så snart det är tillgängligt**.

Item 19

**Jag planerar att aktivt använda videokonsultation i mitt dagliga arbete efter det har implementerats i verksamheten.**

# **Rasch analysis**

## **Performance Expectancy**

Item fit

**Table 9.** Item Fit Statistics PE

| **Item** | **Infit MSQ** | **Infit Thresholds** | **Infit Misfit** | **Relative Location** |
| --- | --- | --- | --- | --- |
| PE1 | 0.729 | [0.628, 1.430] | No misfit | -1.48 |
| PE2 | 0.932 | [0.683, 1.457] | No misfit | -0.22 |
| PE3 | 1.070 | [0.678, 1.406] | No misfit | -0.25 |
| PE4 | 1.242 | [0.641, 1.427] | No misfit | -1.06 |

Note. MSQ values are based on conditional calculations (n = 91 complete cases). Thresholds are based on simulations from 974 datasets.

**Table 10.** Residual Correlations PE

|  | PE1 | PE2 | PE3 | PE4 |
| --- | --- | --- | --- | --- |
| PE1 |  |  |  |  |
| PE2 | -0.02 |  |  |  |
| PE3 | -0.35 | -0.33 |  |  |
| PE4 | -0.12 | -0.49 | -0.22 |  |

Note. Relative cut-off value is 0.116, which is 0.372 above the average residual correlation (-0.256). Residual correlations exceeding the cut-off indicate potential local dependence.

**Table 11.** Principal Component Analysis of Residuals Performance Expectancy

| Component | Eigenvalue | Proportion of Variance |
| --- | --- | --- |
| 1 | 1.59 | 43.4% |
| 2 | 1.50 | 37.0% |
| 3 | 0.89 | 19.3% |
| 4 | 0.01 | 0.30% |

**Figure 3.** Response Category Curve Performance Expectancy

**Table 12.** Item Thresholds and Item Locations Performance Expectancy

| Item | Threshold 1 | Threshold 2 | Threshold 3 | Threshold 4 | Item Location |
| --- | --- | --- | --- | --- | --- |
| PE1 | -2.01 | -2.17 | 1.88 | NA | -0.77 |
| PE2 | -1.14 | -0.23 | 0.04 | 3.38 | 0.49 |
| PE3 | -1.51 | -0.21 | 3.11 | NA | -0.46 |
| PE4 | -2.08 | -1.53 | 2.56 | NA | -0.35 |

Note. Item location represents the mean of the estimated thresholds for each item

DIF for Performance Expectancy: No statistically significant DIF found.

**Figure 4.** Targeting and Person Separation Index for Performance Expectancy

Separation Reliability: 0.7506

Relative measurement uncertainty: WLE-RMU = 0.811 (95% HDCI [0.756, 0.864]) (1000 draws) using package mirt and 50 RMU iterations.

Coefficient alpha (95% CI): 0.77 (0.70,0.84)

**Effort Expectancy**

Itemfit

**Table 13.** Item Fit Statistics for Effort Expectancy

| Item | Infit MSQ | Infit Thresholds | Infit Misfit | Relative Location |
| --- | --- | --- | --- | --- |
| EE1 | 0.974 | [0.672, 1.405] | No misfit | -0.50 |
| EE2 | 0.977 | [0.658, 1.455] | No misfit | 0.21 |
| EE3 | 1.250 | [0.603, 1.479] | No misfit | -1.62 |
| EE4 | 0.667 | [0.652, 1.390] | No misfit | -1.18 |

Note. MSQ values are based on conditional calculations (n = 91 complete cases). Thresholds are derived from 974 simulated datasets.

**Table 14.** Residual Correlations for Effort Expectancy

|  | EE1 | EE2 | EE3 | EE4 |
| --- | --- | --- | --- | --- |
| EE1 |  |  |  |  |
| EE2 | -0.32 |  |  |  |
| EE3 | -0.15 | -0.53 |  |  |
| EE4 | -0.29 | 0.03 | -0.30 |  |

Note. Relative cut-off value is 0.077, which is 0.337 above the average residual correlation (-0.26). Residual correlations exceeding the cut-off indicate potential local dependence.

**Table 15.** Principal Component Analysis of Residuals for Effort Expectancy

| Component | Eigenvalue | Proportion of Variance |
| --- | --- | --- |
| 1 | 1.68 | 48.3% |
| 2 | 1.26 | 31.3% |
| 3 | 0.97 | 18.3% |
| 4 | 0.09 | 2.1% |

**Figure 5.** Response Category Curve for Effort Expectancy

**Table 16.** Item Thresholds and Item Locations for Effort Expectancy

| Item | Threshold 1 | Threshold 2 | Threshold 3 | Item Location |
| --- | --- | --- | --- | --- |
| EE1 | -3.65 | 1.76 | 2.61 | 0.24 |
| EE2 | 0.12 | 1.79 | NA | 0.96 |
| EE3 | -1.41 | -0.34 | NA | 0.44 |
| EE4 | -1.09 | 0.21 | NA | -0.44 |

Note. Item location represents the mean of the estimated thresholds for each item.

DIF Effort Expectancy: No statistically significant DIF found.

**Figure 6**. Targeting and Person Separation Index Effort Expectancy

Separation Reliability: 0.6888

Relative Measurement Uncertainty: WLE-RMU = 0.775 (95% HDCI [0.709, 0.836]) (1000 draws) using package mirt and 50 RMU iterations.

Coefficient alpha (95% CI): 0.76 (0.67,0.84)

## **Social Influence**

Item fit

**Table 17**. Item Fit Statistics for Social Influence

| Item | Infit MSQ | Infit Thresholds | Infit diff | Relative Location |
| --- | --- | --- | --- | --- |
| SI1 | 0.969 | [0.705, 1.312] | No misfit | -0.55 |
| SI2 | 0.889 | [0.663, 1.342] | No misfit | -0.31 |
| SI3 | 1.166 | [0.661, 1.328] | No misfit | -1.00 |
| SI4 | 0.966 | [0.679, 1.347] | No misfit | -0.99 |

Note. MSQ values are based on conditional calculations (n = 91 complete cases). Simulation-based thresholds are derived from 975 simulated datasets.

**Table 18**. Residual Correlations for Social Influence

|  | SI1 | SI2 | SI3 | SI4 |
| --- | --- | --- | --- | --- |
| SI1 |  |  |  |  |
| SI2 | 0.12 |  |  |  |
| SI3 | -0.34 | -0.43 |  |  |
| SI4 | -0.38 | -0.21 | -0.16 |  |

Note. Relative cut-off value is 0.086, which is 0.319 above the average residual correlation (-0.234). Residual correlations exceeding the cut-off indicate potential local dependence.

**Table 19.** Principal Component Analysis of Residuals for Social Influence

| Component | Eigenvalue | Proportion of Variance |
| --- | --- | --- |
| 1 | 1.79 | 45.5% |
| 2 | 1.28 | 32.9% |
| 3 | 0.93 | 21.4% |
| 4 | 0.01 | 0.1% |

**Figure 7.** Response Category Curves for Social Influence

**Table 20.** Item Thresholds and Item Locations for Social Influence

| Item | Threshold 1 | Threshold 2 | Threshold 3 | Item Location |
| --- | --- | --- | --- | --- |
| SI1 | -2.55 | 0.18 | 2.87 | 0.16 |
| SI2 | -2.41 | 0.75 | 2.86 | 0.40 |
| SI3 | -2.91 | 0.15 | 1.91 | -0.28 |
| SI4 | -2.43 | 0.00 | 1.59 | -0.28 |

Note. Item location represents the mean of the estimated thresholds for each item.

DIF for Social Influence: No statistically significant DIF found

**Figure 8.** Targeting and Person Separation Index for Social Influence

Separation Reliability: 0.6441

Relative Measurment Uncertainty: WLE-RMU = 0.734 (95% HDCI [0.659, 0.806]) (1000 draws) using package mirt and 50 RMU iterations.

Coefficient alpha (95% CI): 0.71 (0.62,0.80)

## **Facilitating Conditions**

Item fit

**Table 21.** Item Fit Statistics for Facilitating Conditions

| Item | Infit MSQ | Infit Thresholds | Infit diff | Relative Location |
| --- | --- | --- | --- | --- |
| FC1 | 0.683 | [0.702, 1.320] | 0.019 | -0.72 |
| FC2 | 1.022 | [0.642, 1.424] | No misfit | -0.23 |
| FC3 | 0.837 | [0.675, 1.353] | No misfit | -0.11 |
| FC4 | 1.333 | [0.693, 1.348] | No misfit | -0.14 |

Note. MSQ values are based on conditional calculations (n = 91 complete cases). Simulation-based thresholds are derived from 994 simulated datasets.

**Table 22.** Residual Correlations for Facilitating Conditions

|  | FC1 | FC2 | FC3 | FC4 |
| --- | --- | --- | --- | --- |
| FC1 |  |  |  |  |
| FC2 | 0.05 |  |  |  |
| FC3 | -0.06 | -0.30 |  |  |
| FC4 | -0.41 | -0.54 | -0.16 |  |

Note. Relative cut-off value is 0.076, which is 0.313 above the average residual correlation (-0.237). Residual correlations exceeding the cut-off indicate potential local dependence.

**Table 23.** Principal Component Analysis of Residuals for Facilitating Conditions

| Component | Eigenvalue | Proportion of Variance |
| --- | --- | --- |
| 1 | 1.80 | 50.8% |
| 2 | 1.23 | 28.9% |
| 3 | 0.95 | 19.8% |
| 4 | 0.02 | 0.5% |

**Figure 9.** Response Category Curves for Facilitating Conditions

**Table 24.** Item Thresholds and Item Locations for Facilitating Conditions

| Item | Threshold 1 | Threshold 2 | Threshold 3 | Threshold 4 | Item Location |
| --- | --- | --- | --- | --- | --- |
| FC1 | -2.72 | -0.20 | 1.63 | NA | -0.43 |
| FC2 | -0.34 | 0.46 | NA | NA | 0.06 |
| FC3 | -2.83 | 0.89 | 2.49 | NA | 0.18 |
| FC4 | -2.24 | -1.55 | 1.27 | 3.12 | 0.15 |

Note. Item location represents the mean of the estimated thresholds for each item.

DIF for Facilitating Conditions: No statistically significant DIF found.

**Figure 10.** Targeting and Person Separation Index for Facilitating Conditions

Separation Reliability: 0.638

Relative Measurement Uncertainty: WLE-RMU = 0.775 (95% HDCI [0.708, 0.836]) (1000 draws) using package mirt and 50 RMU iterations

Coefficient alpha (95% CI): 0.76 (0.68,0.84)

## **Behavioral Intention**

Item fit

**Table 25.** Item Fit Statistics for Behavioral Intention

| Item | Infit MSQ | Infit Thresholds | Infit diff | Relative Location |
| --- | --- | --- | --- | --- |
| BI1 | 1.277 | [0.605, 1.431] | No misfit | -0.87 |
| BI2 | 0.574 | [0.657, 1.368] | 0.083 | -1.46 |
| BI3 | 1.176 | [0.721, 1.257] | No misfit | -1.35 |

Note. MSQ values are based on conditional calculations (n = 91 complete cases). Simulation-based thresholds are derived from 995 simulated datasets.

**Table 26.** Residual Correlations for Behavioral Intention

|  | BI1 | BI2 | BI3 |
| --- | --- | --- | --- |
| BI1 |  |  |  |
| BI2 | -0.3 |  |  |
| BI3 | -0.69 | -0.2 |  |

Note. Relative cut-off value is -0.121, which is 0.273 above the average residual correlation (-0.394). Residual correlations exceeding the cut-off indicate potential local dependence.

**Table 27.** Principal Component Analysis of Residuals for Behavioral Intention

| Component | Eigenvalue | Proportion of Variance |
| --- | --- | --- |
| 1 | 1.77 | 69.8% |
| 2 | 1.21 | 29.5% |
| 3 | 0.02 | 0.7% |

**Figure 11.** Response Category Curves for Behavioral Intention

**Table 28.** Item Thresholds and Item Locations for Behavioral Intention

| Item | Threshold 1 | Threshold 2 | Threshold 3 | Item Location |
| --- | --- | --- | --- | --- |
| BI1 | -0.16 | 0.92 | NA | 0.38 |
| BI2 | -0.99 | 0.56 | NA | -0.22 |
| BI3 | -2.46 | -0.31 | 2.45 | -0.11 |

Note. Item location represents the mean of the estimated thresholds for each item.

DIF for Behavioral Intention: No statistically significant DIF found.

**Figure 12.** Targeting and Person Separation Index for Behavioral Intention

Separation Reliability: 0.5579

Relative Measurement uncertainty: WLE-RMU = 0.752 (95% HDCI [0.681, 0.819]) (1000 draws) using package mirt and 50 RMU iterations.

Coefficient alpha (95% CI): 0.80 (0.73,0.87)
